# Structural Diversity and Biological Activities of Fungal Cyclic Peptides, Excluding Cyclodipeptides

**DOI:** 10.3390/molecules22122069

**Published:** 2017-11-27

**Authors:** Xiaohan Wang, Minyi Lin, Dan Xu, Daowan Lai, Ligang Zhou

**Affiliations:** Department of Plant Pathology, College of Plant Protection, China Agricultural University, Beijing 100193, China; wangxiaohan99@126.com (X.W.); linminyi2017@163.com (M.L.); cauxudan@163.com (D.X.); dwlai@cau.edu.cn (D.L.)

**Keywords:** cyclopeptides, fungi, biological activities, occurrence, applications

## Abstract

Cyclic peptides are cyclic compounds formed mainly by the amide bonds between either proteinogenic or non-proteinogenic amino acids. This review highlights the occurrence, structures and biological activities of fungal cyclic peptides (excluding cyclodipeptides, and peptides containing ester bonds in the core ring) reported until August 2017. About 293 cyclic peptides belonging to the groups of cyclic tri-, tetra-, penta-, hexa-, hepta-, octa-, nona-, deca-, undeca-, dodeca-, tetradeca-, and octadecapeptides as well as cyclic peptides containing ether bonds in the core ring have been isolated from fungi. They were mainly isolated from the genera *Aspergillus*, *Penicillium*, *Fusarium*, *Acremonium* and *Amanita*. Some of them were screened to have antimicrobial, antiviral, cytotoxic, phytotoxic, insecticidal, nematicidal, immunosuppressive and enzyme-inhibitory activities to show their potential applications. Some fungal cyclic peptides such as the echinocandins, pneumocandins and cyclosporin A have been developed as pharmaceuticals.

## 1. Introduction

Cyclic peptides (also called cyclopeptides) are cyclic compounds formed mainly by proteinogenic or non-proteinogenic amino acids joined together by amide bonds (or peptide bonds). Cyclic peptides have been isolated from plants [1], fungi [2], bacteria (including actinomycetes) [3], sponges [4], algae [5,6], and mammals [7]. Among these organisms, fungi are well-known producers of a diversity of cyclic peptides with interesting structures and biological activities [8]. Here, we focus on the cyclic peptides derived from fungi, including insect pathogenic fungi, plant pathogenic fungi, soil-derived fungi, marine-derived fungi, plant or insect endophytic fungi, and the macroscopic fungi which we usually call mushrooms. Some of the fungal cyclic peptides are mycotoxins which pose a hazard to animals and plants [2]. Interest in cyclic peptides is due to their significant biological activities, such as antimicrobial, insecticidal, cytotoxic, and anticancer activities, in addition to their important physiological and ecological functions. Therefore, they have the potential to be developed as pharmaceuticals and agrochemicals [9].

Fungal cyclic peptides mainly include cyclic di-, tri-, tetra-, penta-, hexa-, hepta-, octa-, nona-, and decapeptides. Recent special reviews covering the chemical synthesis [10], biosynthesis [11,12], as well as developments and applications (i.e., echinocandins, pneumocandins and cyclosporin A) [13,14] of fungal cyclic peptides are available. In this review, we describe the occurrence, biological activities, structures, and potential applications of the fungal cyclic peptides and their analogs, with the exception of cyclodipeptides (called 2,5-diketopiperazines), which were previously reviewed [15]. Cyclic depsipeptides from organisms including fungi, which are a big group of cyclic peptides in which one or more amino acid is replaced by a hydroxy acid, resulting in the formation of at least one ester bond in the core ring structure, have also been reviewed [16]. The cyclic peptides containing at least one ether bond in the core ring were considered as a special group and included in this review.

## 2. Cyclic Tripeptides

**Figure 1:**
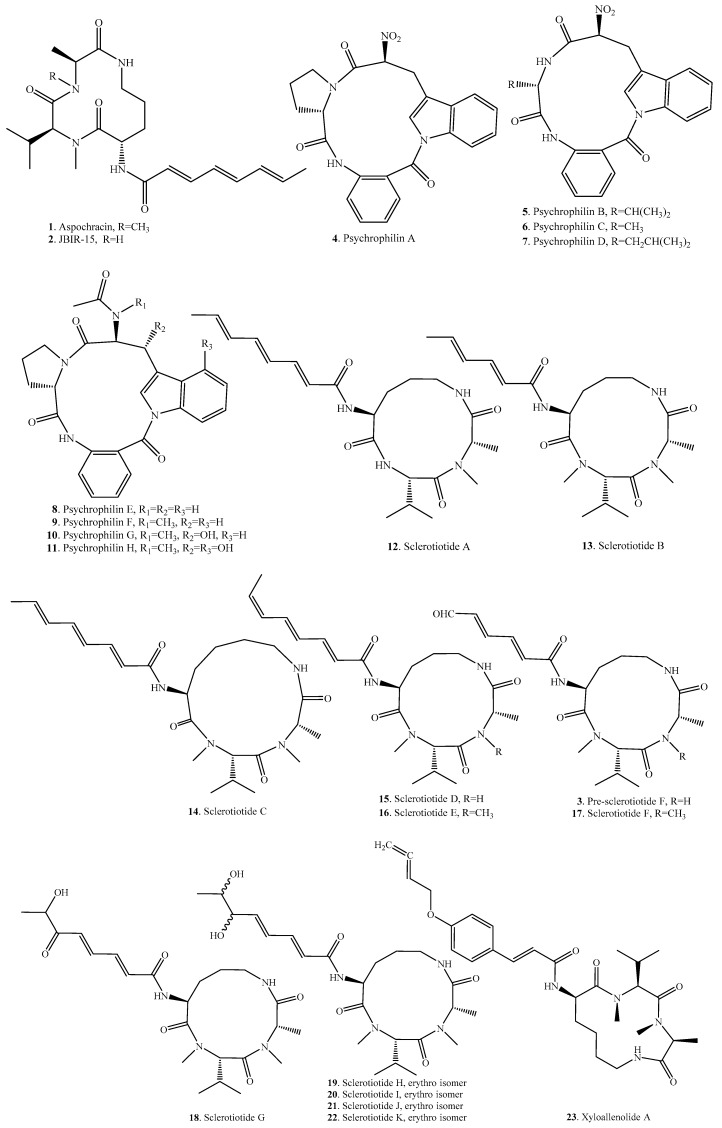
Structures of the cyclic tripeptides isolated from fungi.

Cyclic tripeptides are composed of three amino acid residues in the core ring. They are described in the genera *Aspergillus*, *Penicillium* and *Xylaria*. Cyclic tripeptides exhibit insecticidal [17], cytotoxic [18] and antifungal activities [19]. Their origins, and biological activities are listed in Table 1, and the structures are provided in Figure 1.

Aspochracin (**1**) was the first cyclic tripeptide isolated from fungi in 1969. It was obtained from the culture broth of *Aspergillus ochraceus*, a pathogenic fungus causing muscardine on insects. This compound shows contact toxicity on the first instar larvae as well as the eggs of silkworm [17].

Aspochracin (**1**), JBIR-15 (**2**) and sclerotiotides A–K (**12**–**22**) were obtained from the halotolerant fungus *Aspergillus sclerotiorum* PT06-1. Only sclerotiotides A (**12**), B (**13**), F (**17**) and I (**20**), and JBIR-15 (**2**) show selective antifungal activity against *Candida albicans*, with minimum inhibitory concentration (MIC) values of 7.5, 3.8, 30, 6.7, and 30 μM, respectively [19].

Psychrophilins A–H (compounds **4**–**11**) were isolated from *Aspergillus* and *Penicillium* species [18,20,21,22,23]. Among them, psychrophilin D (**7**) from *Penicillium algidum* show cytotoxic activity [18], psychrophilin E (**8**) from *Aspergillus* sp. shows antiproliferative activity [22], and psychrophilin G (**10**) from *Aspergillus versicolor* ZLN-60 shows lipid-lowering effects [23].

## 3. Cyclic Tetrapeptides

**Figure 2:**
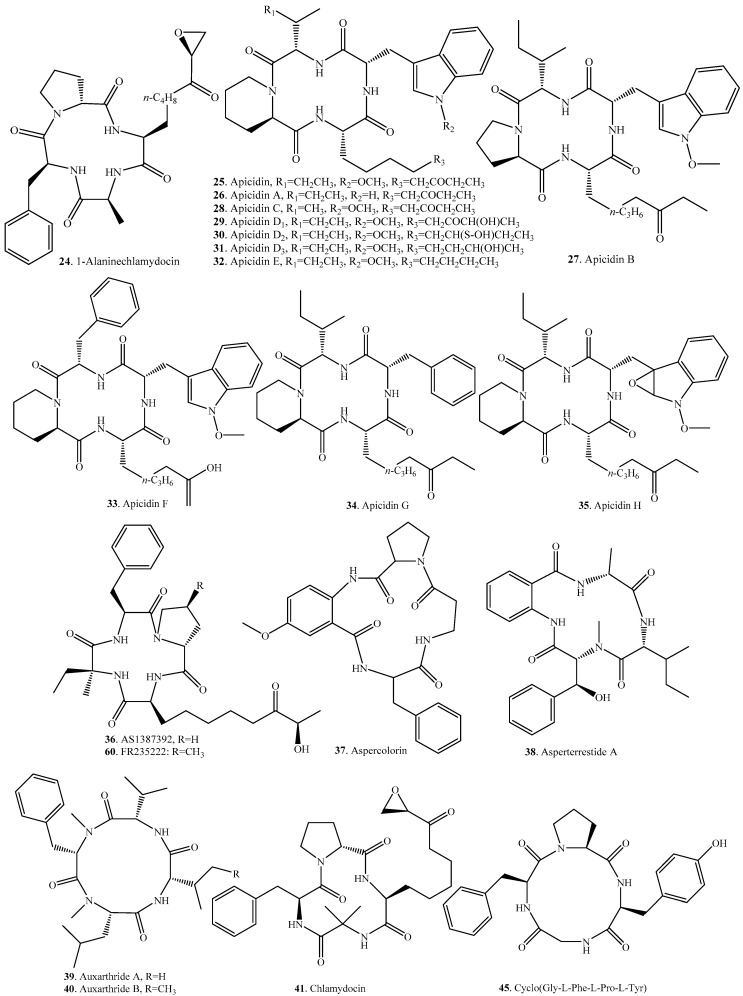

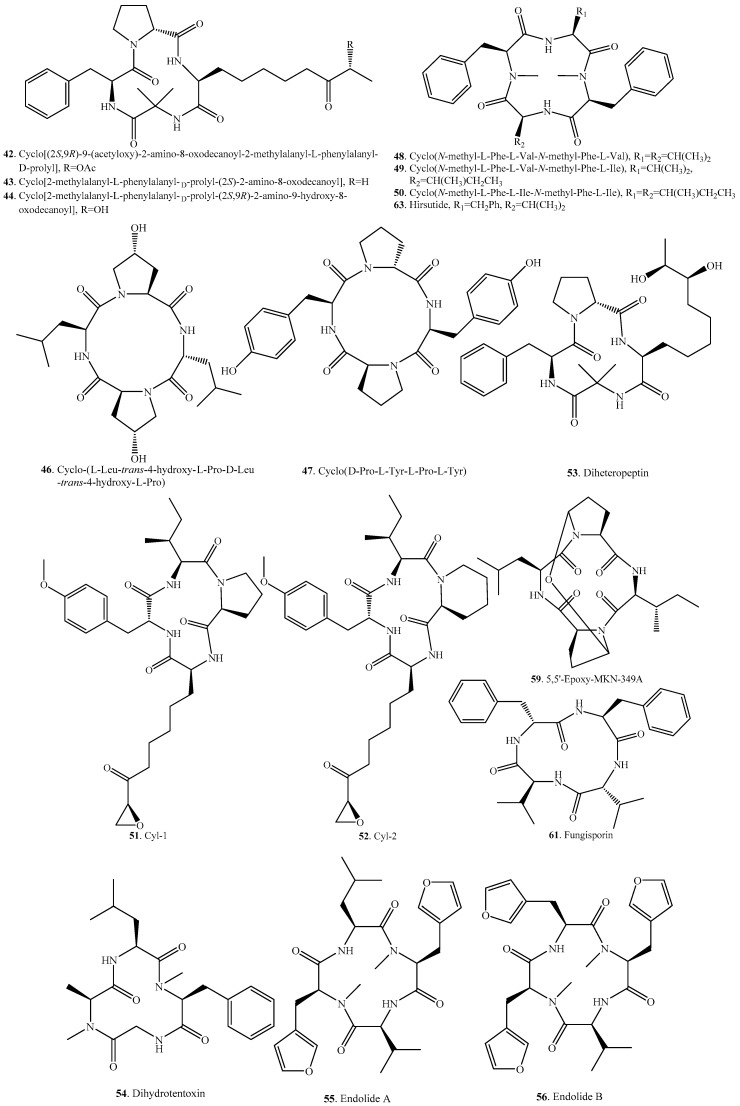

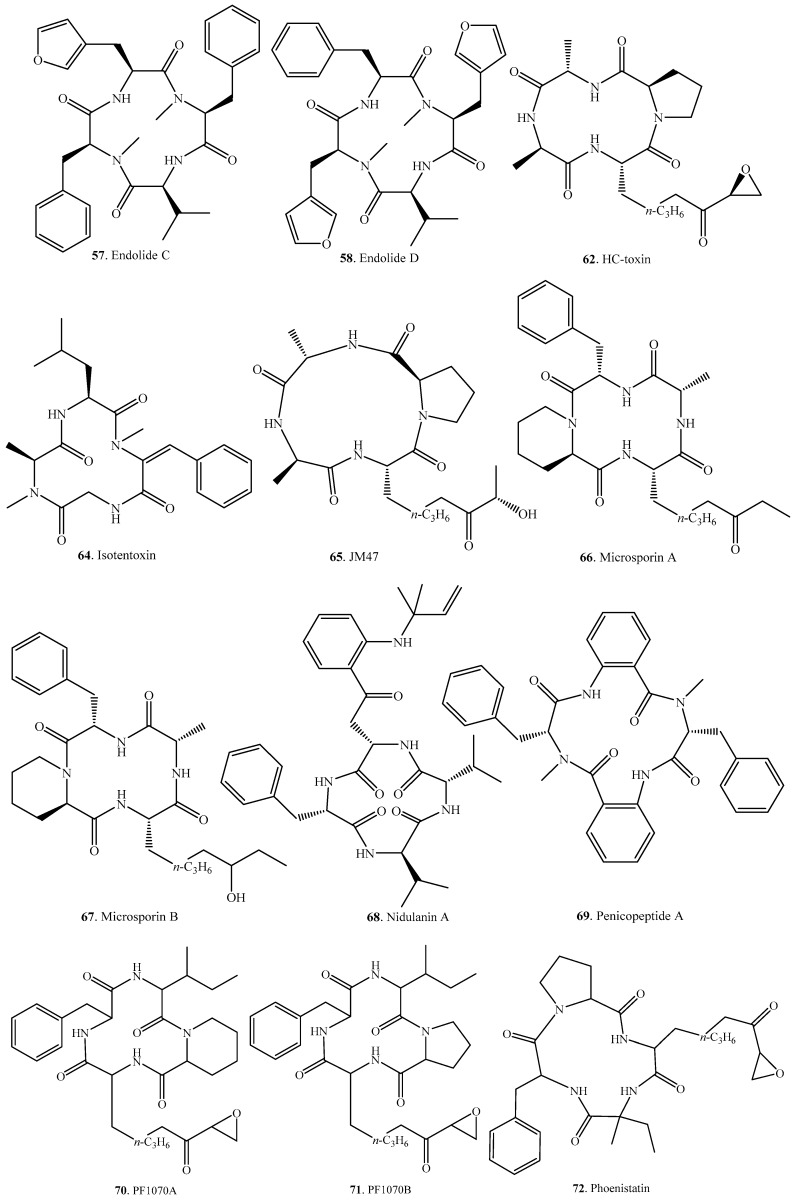

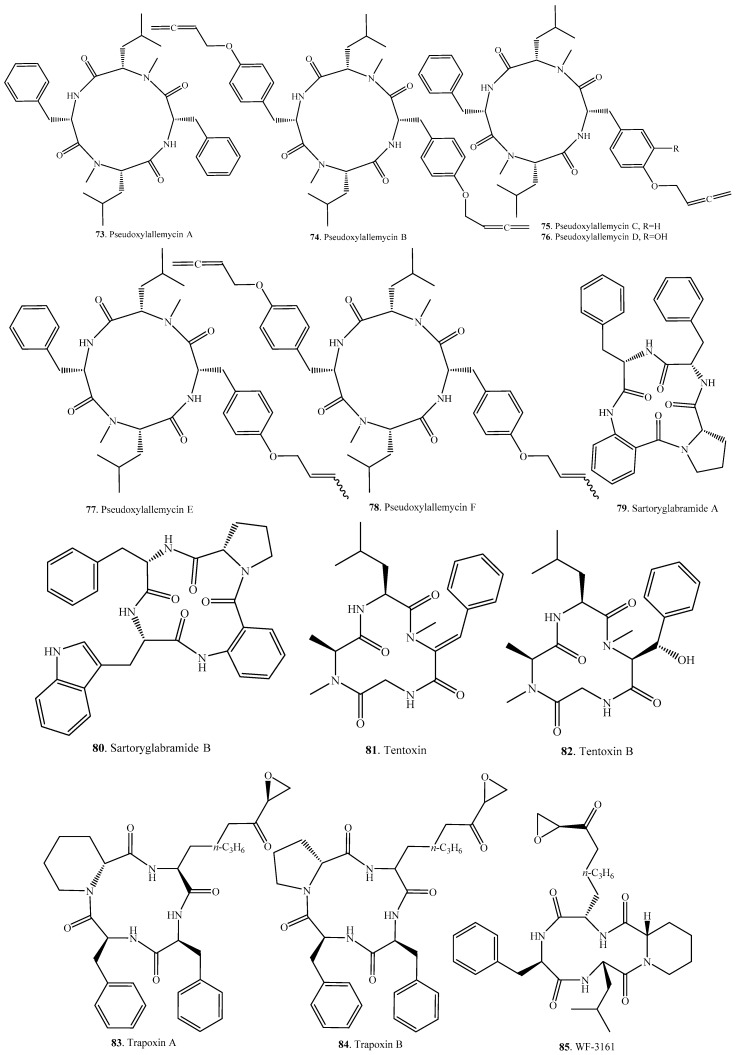
Structures of the cyclic tetrapeptides isolated from fungi.

Cyclic tetrapeptides have been found in many genera such as *Acremonium*, *Alternaria*, *Cylindrocladium*, *Fusarium*, *Helicoma*, *Penicillium*, *Peniophora*, *Phoma*, *Phomopsis*, *Pseudoxylaria*, *Stachylideium*, and *Verticillium*. Their origins and biological activities are listed in Table 2, and the corresponding structures are shown in Figure 2.

Histone deacetylases (HDACs) are important regulators of gene expression and have been implicated as key participants in a variety of diseases. HC-toxins are host-selective toxins and act as inhibitors of HDAC [28]. JM47 (**65**) from a marine *Fusarium* species belongs to the HC-toxin family, and contains a 2-amino-8-oxo-9,10-epoxydecanoic acid residue [29]. Other cyclic tetrapeptides such as 1-alaninechlamydocin (**24**), apicidin (**25**), chlamydocin (**41**), FR235222 (**60**), microsporins A (**66**) and B (**67**), and trapoxins A (**83**) and B (**84**) also show inhibitory activity on HDAC [30,31]. Apicidin analogs (**25**–**35**) from *Fusarium* species show antiprotozoal activity by inhibiting parasite HDAC [32,33,34,35]. These HDAC inhibitors were considered as the potential therapeutics for spinal muscular atrophy [36].

AS1387392 (**36**) from *Acremonium* sp. No. 27082 showed a strong inhibitory effect against mammalian HDAC and T-cell proliferation which suggest**ed** its potential as immunosuppressant [37].

Asperterrestide A (**38**) from the fermentation broth of the marine-derived fungus *Aspergillus terreus* SCSGAF0162 showed cytotoxicity against U937 and MOLT4 hunman carcinoma cell lines and inhibitory effects on influenza virus strains H1N1 and H3N2 [38].

Chlamydocin (**41**) was isolated from *Diheterospora chlantydosporia* [39] and *Penicillium* sp. BCC18034 [40], respectively. This compound showed antimalarial and cytotoxic activities. Three chlamydocin analogs cyclo[(2*S*,9*R*)-9-(acetyloxy)-2-amino-8-oxodecanoyl-2-methylalanyl-l-phenylvalanyl-d-prolyl] (**42**), cyclo[2-methylalanyl-l-phenylalanyl-d-prolyl-(2*S*)-2-amino-8-oxo-decanoyl] (**43**), and cyclo[2-methylalanyl-l-phenylalanyl-d-prolyl-(2*S*,9*R*)-2-amino-9-hydroxy-8-oxodecanoyl] (**44**) were isolated from the culture filtrate of the soil fungus *Peniophora* sp. They all showed plant growth-retarding activity by reducing the height of rice seedlings without blotch and wilting [41].

Three cyclic tetrapeptides cyclo(Gly-l-Phe-l-Pro-l-Tyr) (**45**), cyclo(l-Leu-trans-4-hydroxy-l-Pro-l-Leu-*trans*-4-hydroxy-l-Pro) (**46**) and cyclo(d-Pro-l-Tyr-l- Pro- l -Tyr) (**47**) were isolated from the co-culture broth of two mangrove fungi, *Phomopsis* sp. K38 and *Alternaria* sp. E33. These compounds all had moderate antifungal activity against *Candida albicans*, *Gaeumannomyces graminis*, *Rhzioctonia cerealis*, *Helminthosporium sativum* and *Fusarium graminearum* [42,43].

Three cyclic tetrapeptides cyclo(*N*-methyl-l-Phe-l-Val-*N*-methyl-Phe-l-Val) (**48**), cyclo (*N*-methyl-l-Phe-l-Val-*N*-methyl-l-Phe-l-Ile) (**49**), and cyclo(*N*-methyl-l-Phe-l-Ile-*N*-methyl-l-Phe-l-Ile) (**50**) were isolated from the crude fermentation extract of *Onychocola sclerotica*. They all display inhibitory activity as cardiac calcium channel blockers on wild-type HEK or C1-6-37-3 cells with IC_50_ values of 5.0–7.1 μM [44].

Cyl-1 (**51**) and Cyl-2 (**52**) from the plant fungal pathogen *Cylindrocladium scoparium* exhibited marked inhibition on the growth of rice and lettuce seedlings, especially on their root growth, which suggested that they could be used to inhibit plant growth [45,46].

Microsporins A (**66**) and B (**67**) from the marine-derived fungus *Microsporum* cf. *gypseum* CNL-692 obtained from a sample of the bryozoan *Bugula* sp. collected in the U.S. Virgin Islands. Both compounds showed inhibitory activity on HDAC and demonstrated cytotoxic activity against human colon adenocarcinoma (HCT-116) cells [47].

Penicopeptide A (**69**) was isolated from the endophytic fungus *Penicillium commune* from *Vitis vinifera*. It exhibited significant inhibitory activity against 11β-hydroxysteroid dehydrogenase type 1 (11β-HSD1) in vitro and showed strong binding affinity to recombinant human 11β-HSD1 by microscale thermophoresis assay. Moreover, penicopeptide A (**69**) decreased the lipid droplet accumulation associate with the inhibiton of 11β-HSD1 activity of 3T3-L1 cells in vivo [48].

Pseudoxylallemycins A–F (**73**–**78**) were isolated from the termite-associated fungus *Pseudoxylaria* sp. X802. Pseudoxylallemycins A–D (**73**–**76**) showed antibacterial activity against Gram-negative human-pathogenic *Pseudomonas aeruginosa* and antiproliferative activity against human umbilical vein endothelial cells and K-562 cell lines [49].

Tentoxin (**81**) and its derivatives dihydrotentoxin (**54**) and isotentoxin (**64**) were important mycotoxins from *Alternaria* spp. [50]. Tentoxin B (**82**) was isolated from the marine-derived fungus *Phoma* sp. from the giant jellyfish *Nemopilema nomurai*. This compound showed weak suppressive effect on the production of nitric oxide in murine macrophage cells [51].

Trapoxins A (**83**) and B (**84**) from *Helicoma ambiens* RF-1023 exhibited detransformation activity against *v-sis* oncogene-transformed NIH3T3 cells (*sis*/NIH3T3) [52]. In addition, trapoxin A (**83**) showed HDAC inhibitory activity [31]. Both compounds displayed their potential as antitumor agents.

## 4. Cyclic Pentapeptides

**Figure 3:**
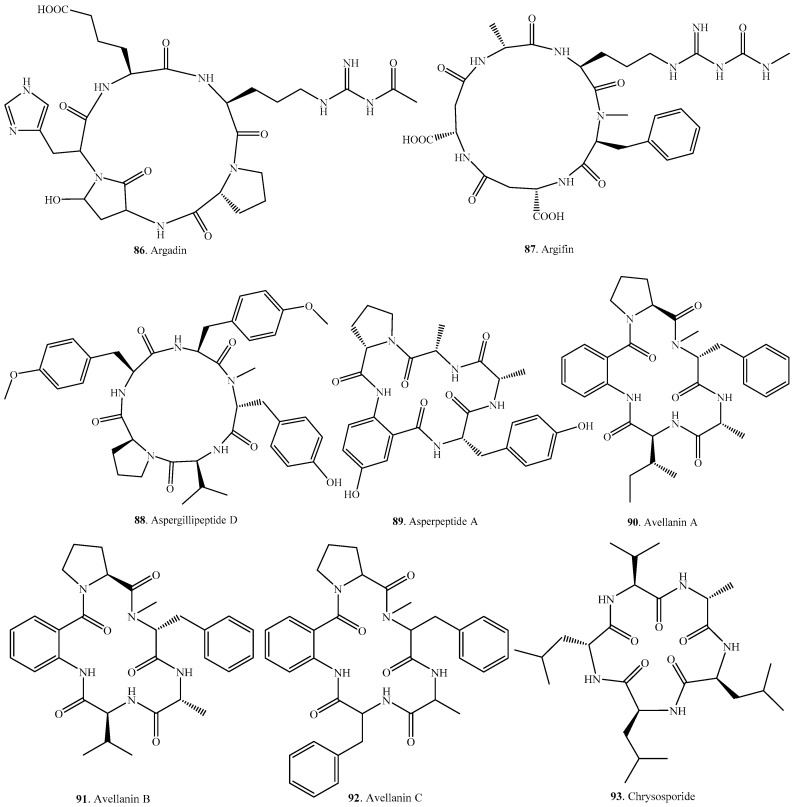

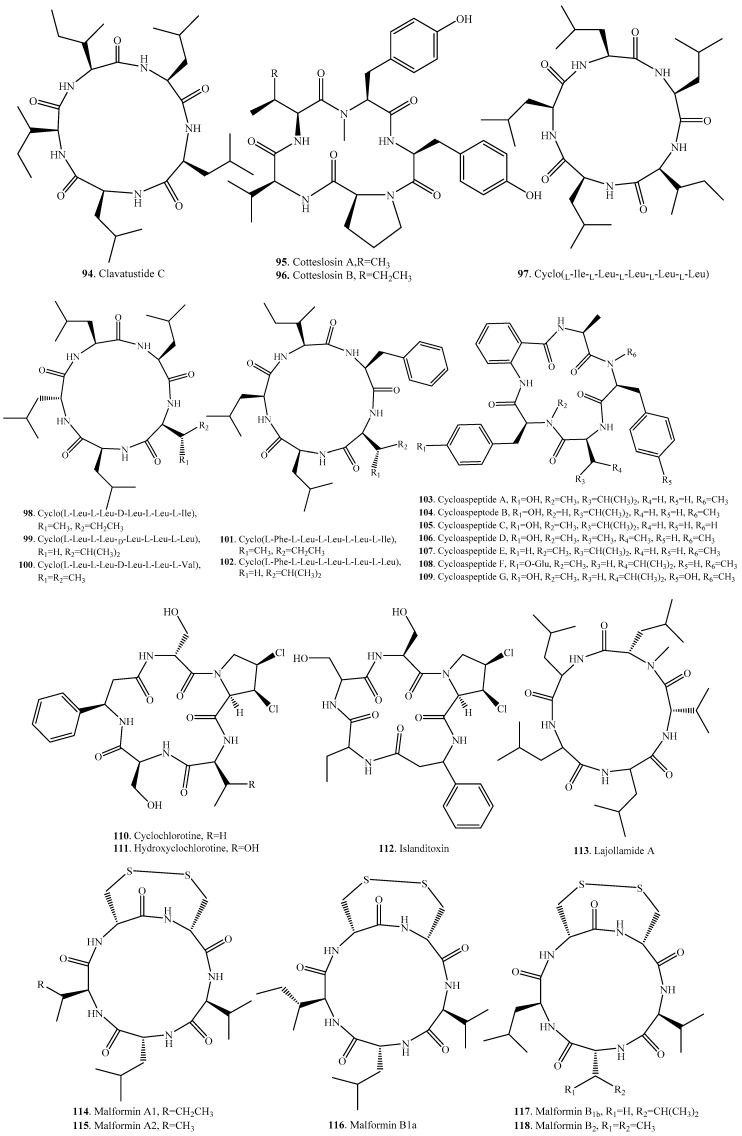

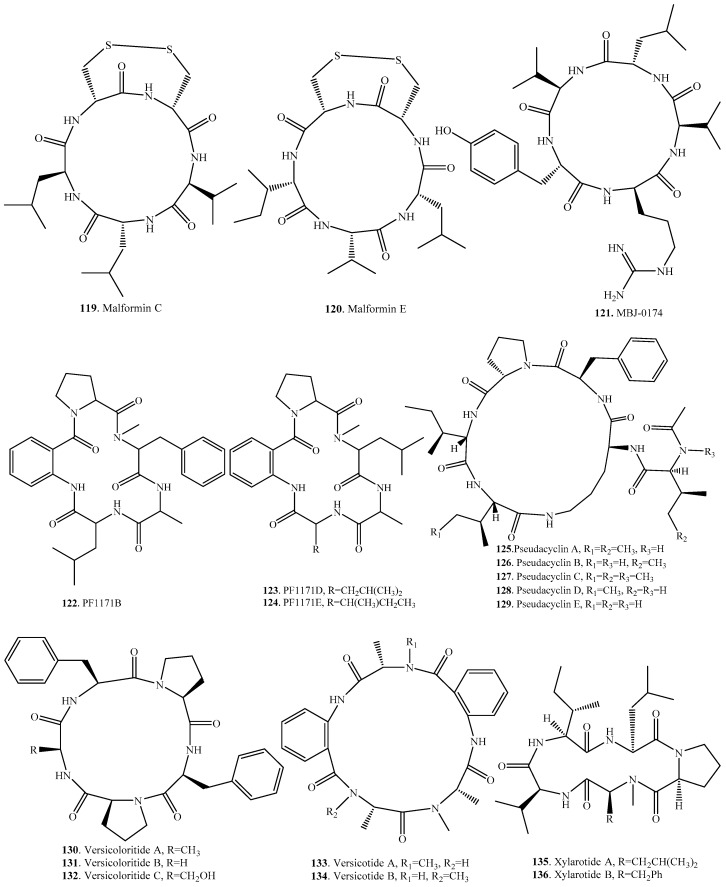
Structures of the cyclic pentapeptides isolated from fungi.

Cyclic pentapeptides have been mainly isolated from the genera of *Aspergillus*, *Fusarium*, *Hamigera*, *Penicillum*, *Pseudallescheria* and *Xylaria*. Their distributions in fungi, and biological activities are listed in Table 3, and the structures are provided in Figure 3.

Argadin (**86**) and argifin (**87**) produced by *Clonostachys* sp. FO-7314 and *Gliocladium* sp. FTD-0668, respectively, were identified in screening as chitinase inhibitors [81,82]. Chitinases play key roles in organisms, ranging from bacteria to humans. There is a need for specific, potent inhibitors to probe the function of these chitinases in different organisms. Such molecules could also provide leads for the development of chemotherapeuticals with fungicidal, insecticidal, or anti-inflammatory potential. Argadin (**86**) and argifin (**87**) could be used as the leads for the development of novel chitinase inhibitors [83].

Aspergillipeptide D (**88**) was isolated from the marine gorgonian-derived fungus *Aspergillus* sp. SCSIO 41501. It showed evident antiviral activity against herpes simplex virus type 1 (HSV-1) with a median inhibitory concentration (IC_50_) value of 9.5 μM under their non-cytotoxic concentration against a Vero cell line, and also had antiviral activity against acyclovir-resistant clinical isolates of HSV-1 [84].

Cycloaspeptides A–F (**103**–**108**) are anthranilic acid (ABA)-containing cyclic pentapeptides. Among them, cycloaspeptides A–C (**103**–**105**) were isolated from *Aspergillus* sp. NE-45 [85], cycloaspeptide D (**106**) with antiplasmodial activity from *Penicillium algidum* [18] and the psychrotolerant fungus *Penicillium ribeum* [20], cycloaspeptide E (**107**) with insecticidal activity from several *Penicillia* species and a *Tricothecium* strain [86], and cycloaspeptides F (**108**) and G (**109**) from *Isaria farinosa* [87]. The presence of a glucose unit in cycloaspeptide F (**108**) was very rare in the isolated cyclic peptides.

Seven malformins (**114**–**120**) were isolated from a few *Aspergillus* species [88,89,90,91]. Malformin A1 (**114**) from *Aspergillus niger* and *Aspergillus tubingensis* showed malformation activity in the corn root, and cytotoxic activity, and anti-TMV activity [89,90,92,93]. Malformin C (**119**) from *Aspergillus niger* had potent cell growth inhibition activity [94], but its therapeutic index was too low to be an anti-cancer drug [95].

Pseudacyclins A–E (**125**–**129**) were isolated from *Pseudallescheria boydii* which is an emerging fungal pathogen causing fatal infections in both immunocompromised and immunocompetent hosts. Among them, pseudacyclin A (**125**) exhibited immunosuppressive activity [96].

## 5. Cyclic Hexapeptides

**Figure 4:**
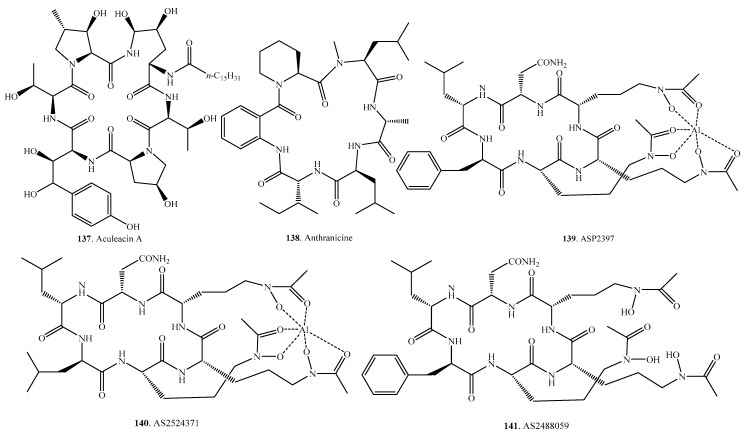

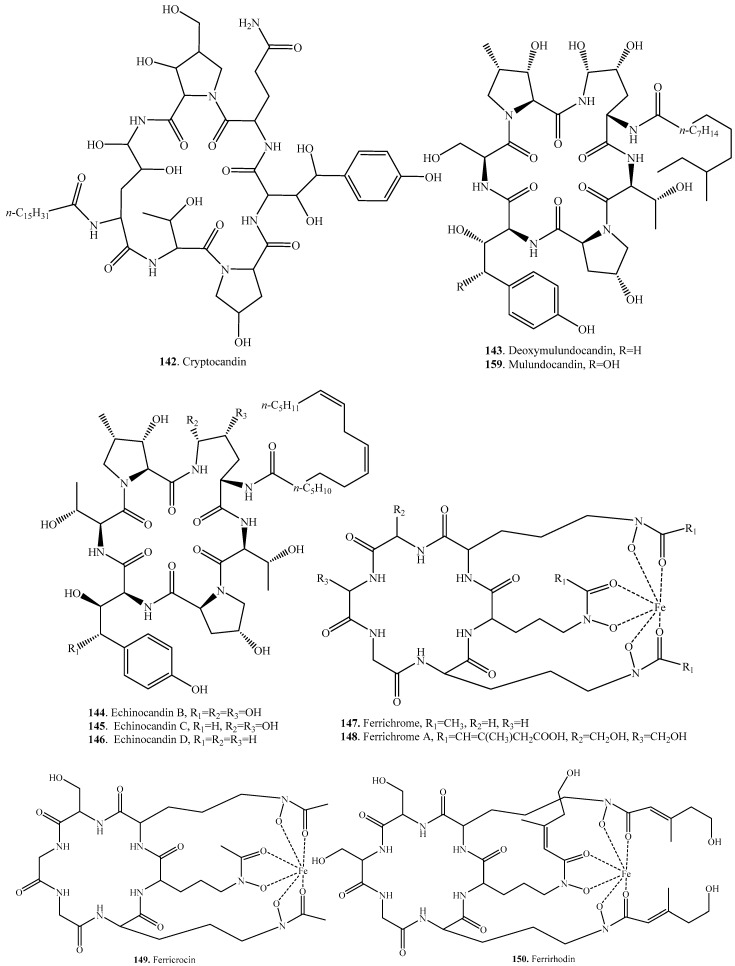

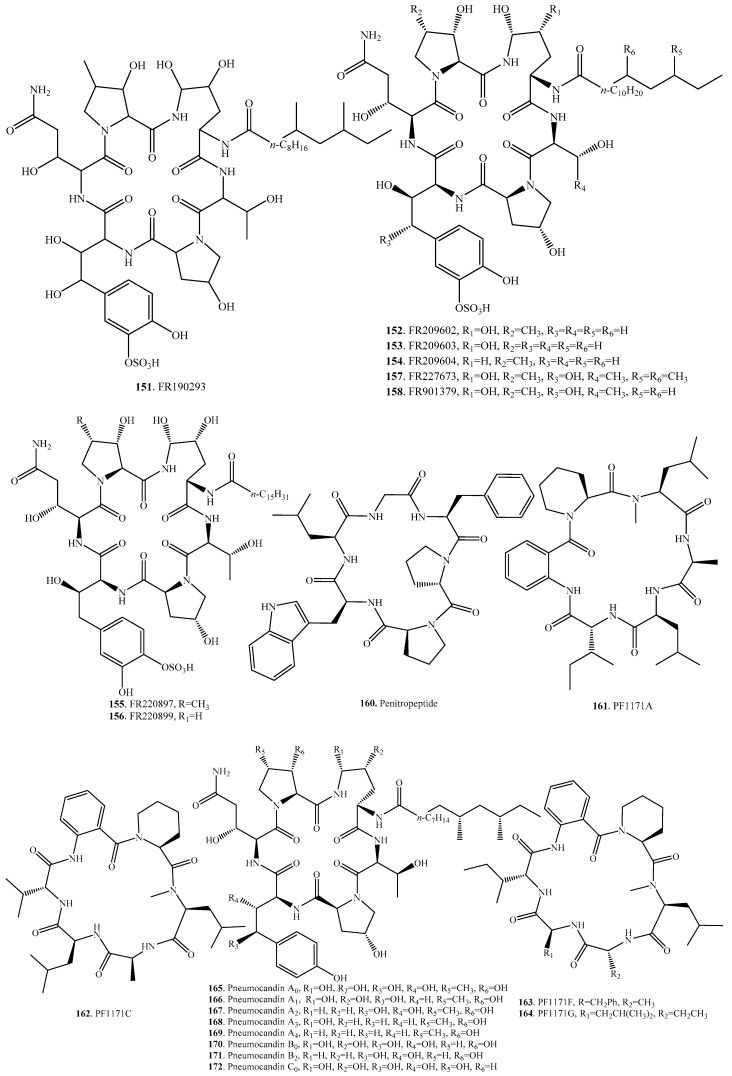

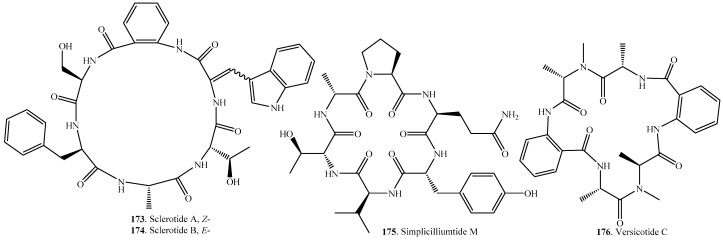
Structures of the cyclic hexapeptides isolated from fungi.

About 40 cyclic hexapeptides have been isolated from fungi. Their distributions in fungi, and biological activities are shown in Table 4, and the structures are provided in Figure 4.

Aculeacin A (**137**) was isolated from *Aspergillus aculeatus* M-4214. This compound showed marked antifungal activity by inhibiting synthesis of β-1,3-glucan [117,118].

The echinocandins have emerged as first-line antifungal agents for many *Candida* and *Aspergillus* infections. They have a unique mechanism of action by inhibiting the synthesis of β-1,3-d-glucan, a key component of the fungal cell wall. Caspofungin was the first echinocandin antifungal agent to become licensed for treatment of invasive fungal infecitons [119]. Caspofungin has been used in immunocompromised children and neonates with invasive fungal infections [120]. The second commercial antifungal agent in the echinocandin series was micafungin which has been used worldwide in chemotherapy for life-threatening fungal infections [121].

Mulundocandin (**159**) and deoxymulundocandin (**143**) were isolated from *Aspergillus sydowii* [122] and *Aspergillus mulundensis* [123], respectively. Both compounds showed antifungal activity against yeasts and filamentous fungi.

PF1171A (**161**) and PF1171C (**162**) were isolated from an unidentified ascomycete OK-128. Both compounds showed paralytic activity against silkworms [124]. PF1171C (**162**) was reisolated as similanamide (**162**) from the marine sponge-associated fungus *Aspergillus similanensis* KUFA 0013. It showed weak cytotoxic activity against the cancer cell lines [125,126].

Pneumocandin anlogues (**165**–**172**) were isolated from *Zalerion arboricola*. They all showed anticandial activity by inhibiting 1,3-β-d-glucan synthesis [127,128].

Sclerotides A (**173**) and B (**174**) were isolated from *Aspergillus sclerotiorum* PT06-1. They all showed moderate antifungal activity against *Candida albicans*. In addition, sclerotide B (**174**) exhibited antibacterial and cytotoxic activities [129].

## 6. Cyclic Heptapeptides

**Figure 5:**
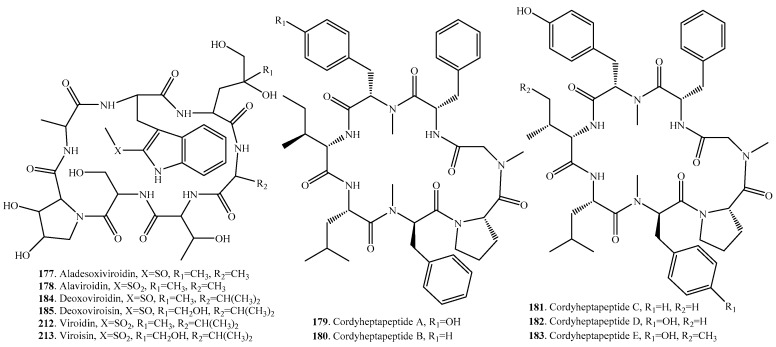

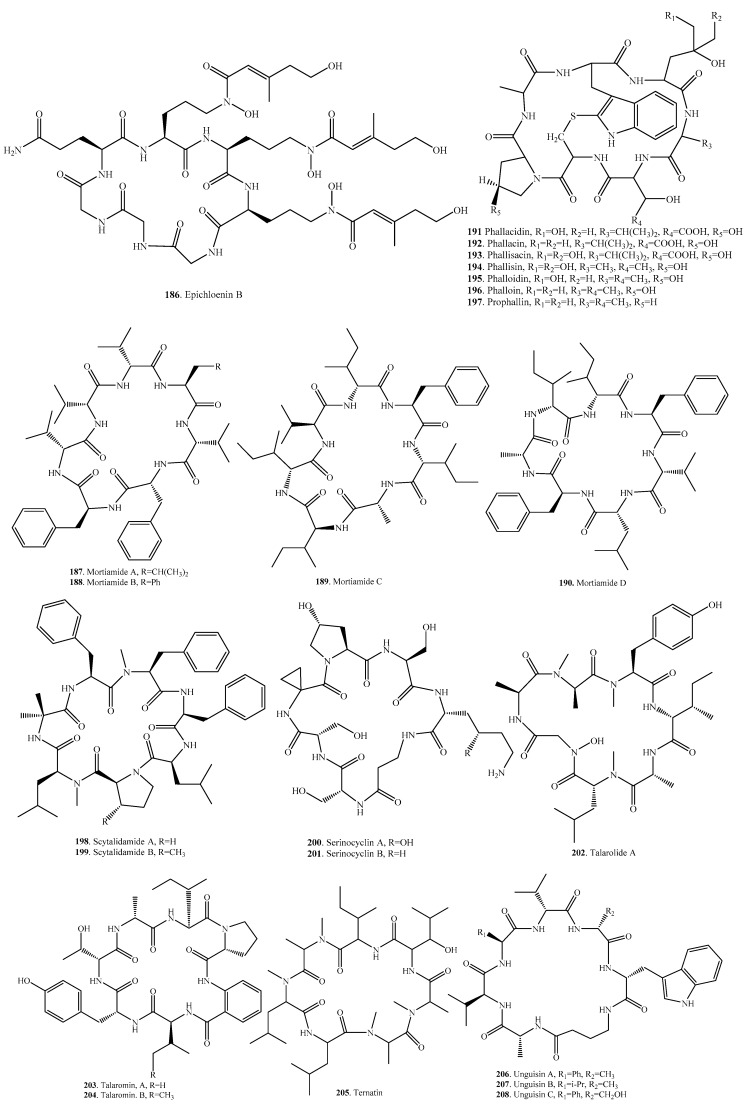

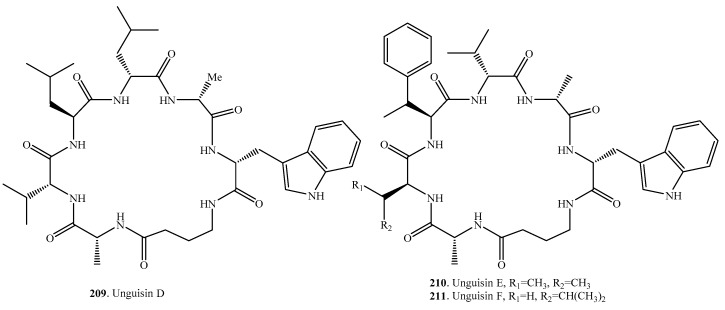
Structures of the cyclic heptapeptides isolated from fungi.

There are 37 cyclic heptapeptides isolated from fungi. Their distributions in fungi, and biological activities are listed in Table 5, and the structures are shown in Figure 5.

Cordyheptapeptides A (**179**) and B (**180**) were isolated from *Cordyceps* sp. which is an insect fungal pathogen [147,148]. Cordyheptapeptides C (**181**), D (**182**) and E (**183**) were isolated from the marine-derived fungus *Acremonium persicinum* SCSIO 115 [149]. Cordyheptapeptides A (**179**), B (**180**), C (**181**) and E (**183**) all showed cytotoxic activity [148,149].

Phallotoxins (**191**–**197**) were bicyclic heptapeptides cross-linked by the 2’-bound sulfur group from the poisonous mushroom *Amanita phalloides*. Seven phallotoxins: phallacidin (**191**), phallacin (**192**), phallisacin (**193**), phallisin (**194**), phalloidin (**195**), phalloin (**196**), and prophallin (**197**), have been identified so far [150]. Phallotoxins were shown to be ribosomally synthesized and posttranslationally modified peptides (RiPPs) [151].

Scytalidamides A (**198**) and B (**199**) were isolated from *Acremonium* sp. [152] and *Scytalidiu* sp. [153], respectively. Both compounds showed cytotoxic activity toward HCT-116 human colon adenocarcinoma with IC_50_ values of 2.7 and 11.0 μM, respectively [153].

Unguisins A–D (**206**–**209**) were isolated from *Emericella unguis* [154,155], unguisin E (**210**) from *Aspergillus* sp. AF119 [156] and *Mucor irregularis* [157], and unguisin F (**211**) from *Mucor irregularis* [157]. Only unguisins A (**206**) and B (**207**) were screened to display moderate antibacterial activity [154,155]. Further investigation showed that unguisin A (**206**) was an anion receptor with high affinity for phosphate and pyrophosphate [158].

Virotoxins (**177**,**178**,**184**,**185**,**212**,**213**) are mono-cyclic heptapeptides containg a tryptophan residue substituted at position 2 of the indol ring by a sulfur atom. A series of virotoxins such as aladesoxiviroidin (**177**), alaviroidin (**178**), deoxoviroidin (**184**), deoxoviroisin (**185**), viroidin (**212**), and viroisin (**213**) have been identified from the mushrooms *Amanita phalloides* and *Amanita virosa* so far. They were toxic to animals [150,159].

## 7. Cyclic Octapeptides

**Figure 6:**
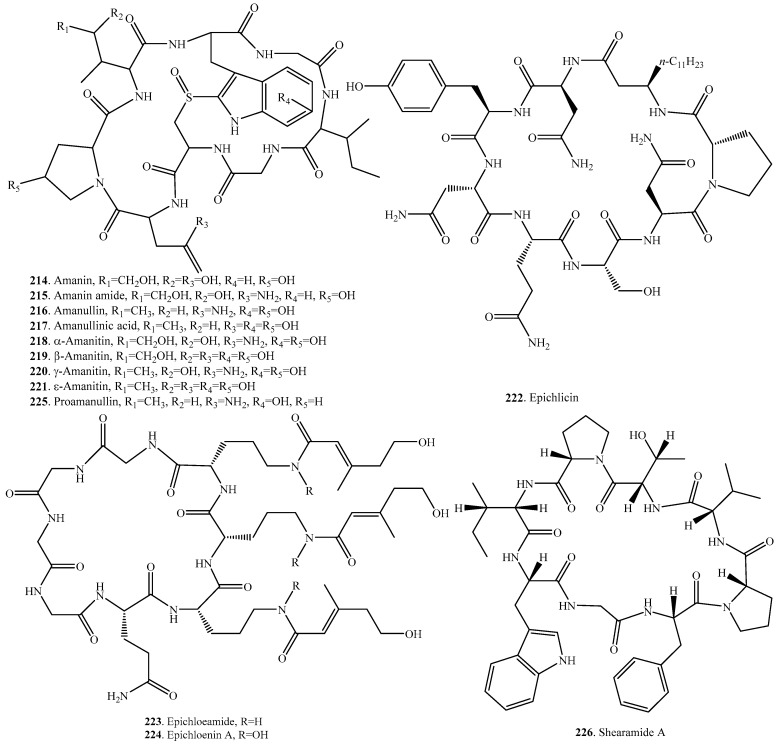
Structures of the cyclic octapeptides isolated from fungi.

The distributions and biological activities of fungal cyclic octapeptides are listed in Table 6, and the structures are shown in Figure 6.

Fungal cyclic octapeptides mainly include the amatoxins, which are bicyclic octapeptides distributed in the poisonous mushroom *Amanita phalloides*. Nine amatoxin analogs amanin (**214**), amanin amide (**215**), amanullin (**216**), amanullinic acid (**217**), α-amanitin (**218**), β-amanitin (**219**), γ-amanitin (**220**), ε-amanitin (**221**), and proamanullin (**225**) have been identified [150]. The most important biochemical effect of amatoxins is the inhibition of RNA polymerases (especially polymerase II). This interaction leads to a tight complex, and the inhibition is of a non-competitive type. Non-mammalian polymerases show little sensitivity to amanitins. The amatoxins cause necrosis of the liver, also partly in the kidney, with the cellular changes causing the fragmentation and segregation of all nuclear components. Various groups of somatic cells of emanation resistance have been isolated, including from a mutant of *Drosophila melanogaster* [150].

Amatoxins are synthesized as proproteins on ribosomes, and are considered as ribosomally synthesized and post-translationally modified peptides (RiPPs). The proproteins are 34–35 amino acids in length and do not have predicted signal peptides [151].

Epichlicin (**222**) was isolated from the endophytic fungus *Epichloe typhina* from the timothy plant (*Pheum pretense*). This compound exhibited inhibitory activity toward the spore germination of *Cladosporium phlei*, a fungal pathogen of the timothy plant at an IC_50_ value of 22 nM [166].

Both epichloeamide (**223**) and epichloenin A (**224**) were identified from the endophytic fungus *Epichloe festucae* and endophyte-infected *Lolium perenne* [160].

Shearamide A (**226**) was isolated from the ascostromata of *Eupenicillium shearii*. This compound showed insecticidal effect against *Helicoverpa zea* larvae [167].

## 8. Cyclic Nonapeptides

**Figure 7:**
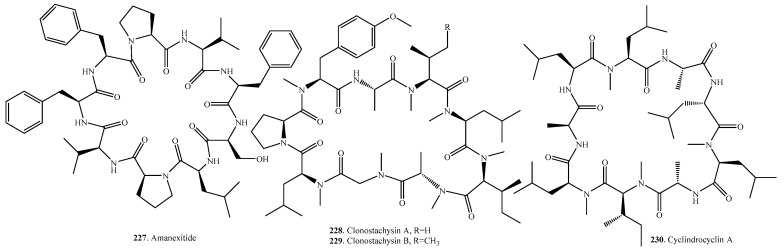
Structures of the cyclic nonapeptides isolated from fungi.

Only four cyclic nonapeptides have been identified in fungi, with their occurrence, biological activities and structures shown in Table 7 and Figure 7, respectively. Amanexitide (**227**) was isolated from the fruiting bodies of *Amanita exitialis*, a poisonous mushroom endemic in China [168]. Both clonostachysins A (**228**) and B (**229**) were obtained from a marine sponge-derived fungus *Clonostachys rogersoniana* strain HJK9. They exhibited a selectively inhibitory activity on the dinoflagellate *Prorocentrum micans* at 30 μM [170]. Cylindrocyclin A (**230**) from *Cylindrocarpon* sp. exhibited cytotoxic activity against six cell lines with IC_50_ values ranging from 11 to 53 μM [171].

## 9. Cyclic Decapeptides

**Figure 8:**
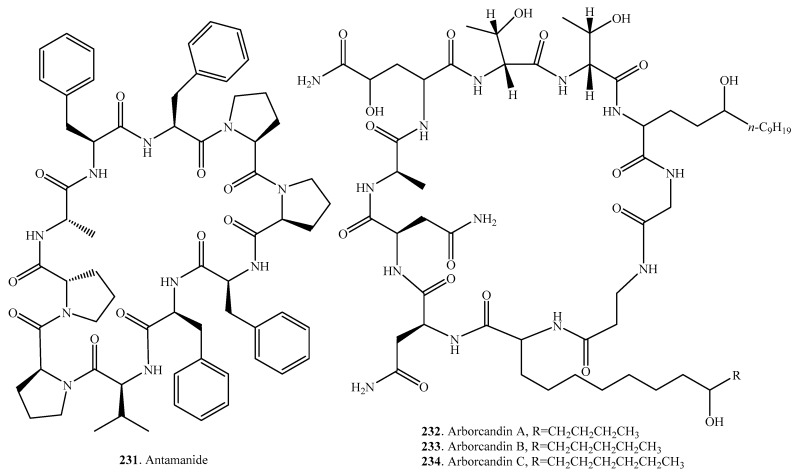

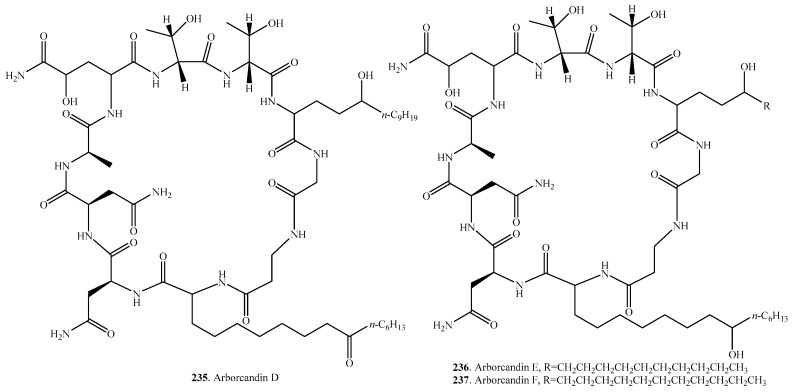
Structures of the cyclic decapeptides isolated from fungi.

A total of seven cyclic decapeptides were identified in fungi. Their distributions, biological activities and structures are shown in Table 8 and Figure 8.

Antamanide (**231**) derived from the fungus *Amanita phalloides* inhibited the mitochondrial permeability transition pore, a central effector of apoptosis induction, by targeting the pore regulator cyclophillin D [172].

Arborcandins A–F (**232**–**237**) were isolated from the culture broth of an unidentified filamentous fungus SANK 17397. They possessed potent 1,3-β-glucan synthase inhibitory activity [173,174].

## 10. Cyclic Undecapeptides

**Figure 9:**
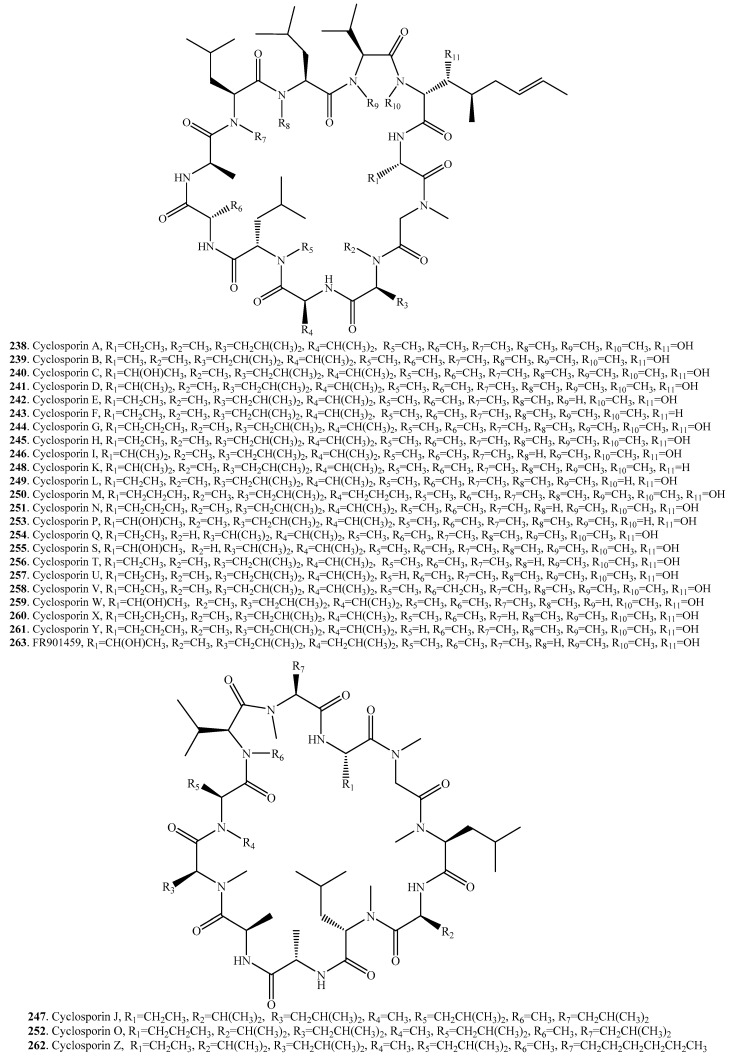
Structures of the cyclic undecapeptides isolated from fungi.

Twenty-six cyclosporin analogs were identified in fungi. They belong to the cyclic undecapeptide group, and their distributions, biological activities and structures are shown in Table 9 and Figure 9, respectively.

Among them, cyclosporin A (**238**) has received the most meticulous attention owing to its immunosuppressive and antifungal activity [176]. Cyclosporin A (**238**) can be produced by a series of fungi such as *Aspergillus fumigatus* [177], *Aspergillus terreus* [178], *Beauveria nivea* [179], *Fusarium oxysporum* [180], *Trichoderma polysporum* [181], *Tolypocladium inflatum* [182], and *Tolypocladium* sp. [183]. It was mainly produced by various types of fermentation techniques using *Tolypocladium inflatum*. It has a variety of biological activities including immunosuppressive, anti-inflammatory, antifungal and antiparasitic properties. The mechanism of action has been considered as the phosphatase activity inhibition of calcineurin, and production of IL-2 and other cytokines [176].

FR901459 (**263**), a derivative of cyclosporin A (**238**), was discovered in the fermentation broth of *Stachybotrys chartarum* No. 19392 (isolated from soil collected in Tokyo Prefecture, Japan). This compound showed antifungal activity, and was capable of prolonging the survival time of skin allografts in rats with one-third the potency of cyclosporin A [184]. It also prevented mitochondrial swelling and protected against delayed neuronal cell death [185].

## 11. Cyclic Dodecapeptides

**Figure 10:**
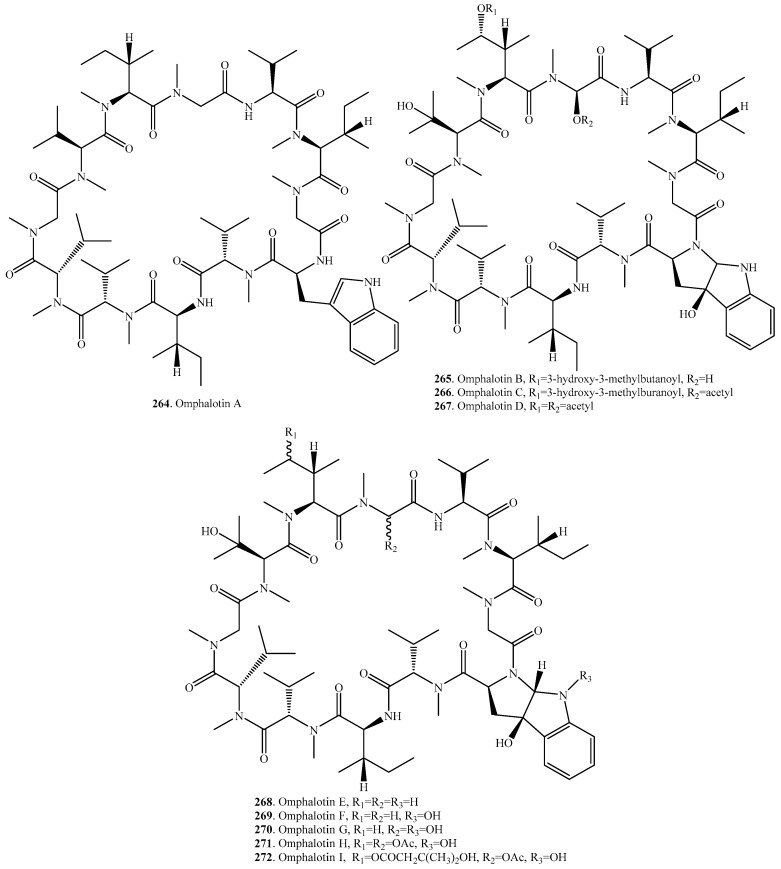
Structures of the cyclic dodecapeptides isolated from fungi.

Omphalotin analogs are the only cyclic dodecapeptides idolated from fungi so far. Omphalotins (**264**–**272**) were isolated from the basidiomycetes mushroom *Omphalotus olearius*. These compounds are toxic to humans and animals. Their distributions, biological activities and structures are shown in Table 10 and Figure 10, respectively. Nine cyclic dodecapeptides omphalotins A–I (**264**–**272**) with nematicidal activity were identified in the mushroom *O. olearius* [191,192]. They have been considered as the potent nematicidal agents that seemed to be highly selective for *Meloidogyne incognita* [193]. Omphalotins were recently identified as the ribosomally synthesized and posttranslationally modified peptides (RiPPs). A self-sacrificing *N*-methyltransferase was shown to be the precursor of omphalotin A (**264**) [194,195,196].

## 12. Cyclic Tetradecapeptides

**Figure 11:**
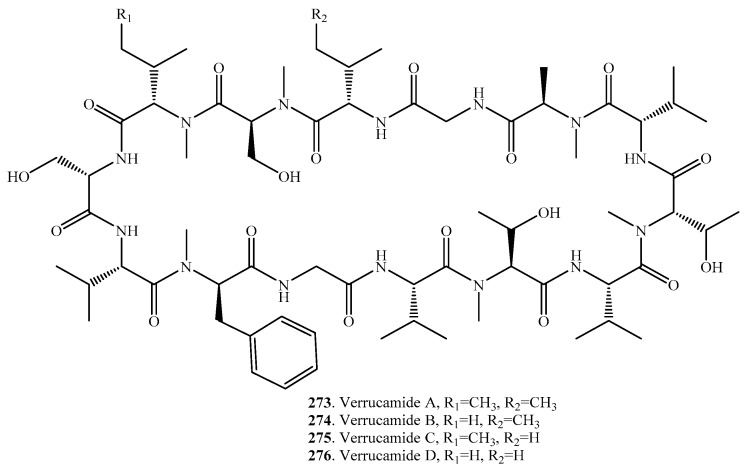
Structures of the cyclic tetradecapeptides isolated from fungi.

Only four cyclic tetradecapeptides, verrucamides A–D (**273**–**276**), were identified from the plant pathogenic fungus *Myrothecium verrucaria* that attacked important crop plants and weeds. Their distributions, biological activities and structures are shown in Table 11 and Figure 11, respectively. They were screened to show antibacterial activity against *Staphylococcus aureus* [200].

## 13. Cyclic Octadecapeptides

**Figure 12:**
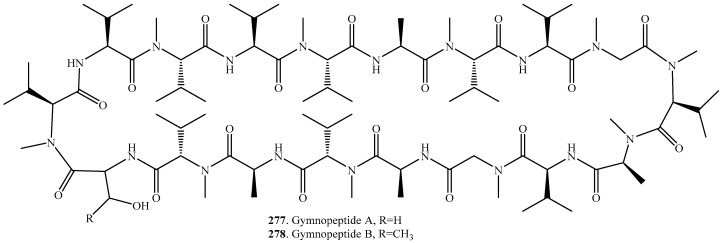
Structures of the cyclic octadecapeptides isolated from fungi.

Only two highly *N*-methylated cyclic octadecapeptides namely gymnopeptides A (**277**) and B (**278**) were isolated from the mushroom *Gymnopus fusipes* (Table 12 and Figure 12). They exhibited striking antiproliferative activity on several human cancer cell lines [201]. So far, gymnopeptides A (**277**) and B (**278**), constituted by 18 monomers, are the largest cyclic peptides to be isolated from fungi. Fortunately, the total synthesis of gymnopeptides A (**277**) and B (**278**) has been achieved [202]. Further biological studies are needed to clarify the aspects of absorption and metabolism of gymnopeptides A (**277**) and B (**278**) after consumption of the fruiting bodies by humans, as well as to explore the potential role of these metabolites in the parasitic lifestyle of the mushroom *G. fusipes*.

## 14. Cyclic Peptides Containing Ether Bonds in the Core Ring

**Figure 13:**
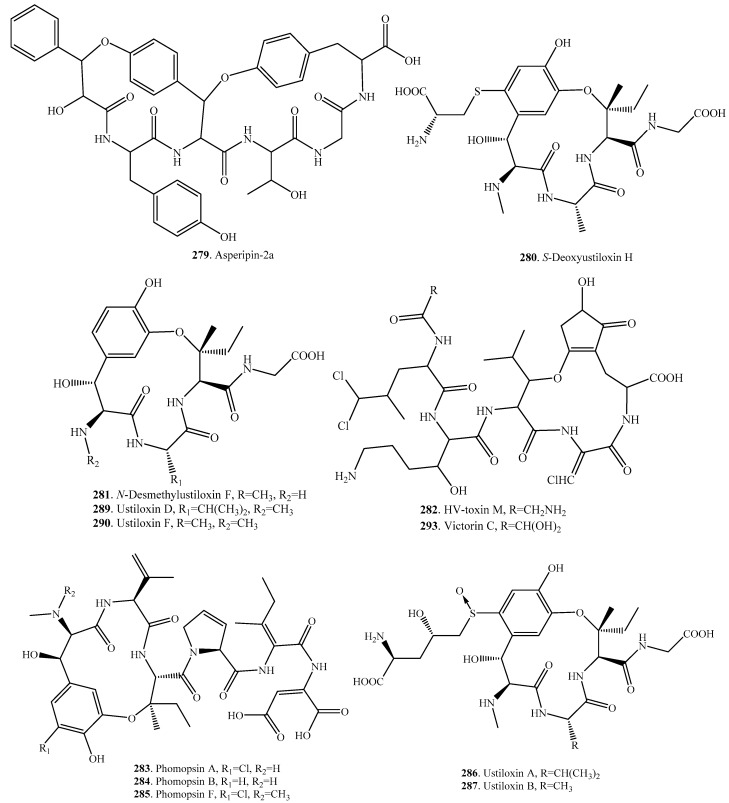

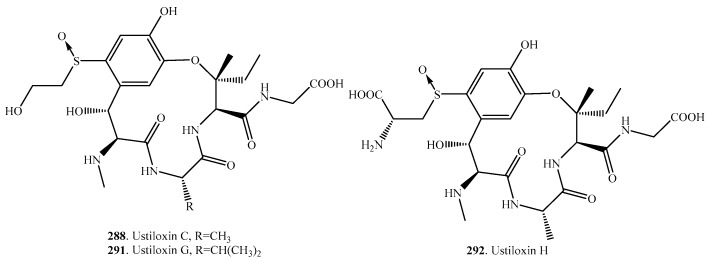
Structures of the fungal cyclic peptides containing ether bonds in the core ring.

This group of cyclic peptides contained at least one ether linkage except the normal amide and carbon-carbon bonds in the core ring of each molecule. Their distributions, biological activities and structures are shown in Table 13 and Figure 13, respectively.

Asperipin-2a (**279**) containing five amino acids was isolated from *Aspergillus flavus*. It was a bicycle peptide containing two ether linkages that was converted from the repeated sequence containing aromatic residues [203].

HV-toxin M (**282**), with phytotoxic activity, was isolated from the plant pathogenic fungus *Cochliobus victoriae* (previously as *Helminthosporium victoria*) from oat (*Avena sativa*). This compound had three amino acids in the core rings, and two amino acids linked and attached as the side chains [204].

Phomopsins A (**283**), B (**284**) and F (**285**) were isolated from *Phomopsis leptostromiformis* [205,206] and *Diaporthe toxica* [207]. They have a structure similar to HV-toxin M (**282**). Phomopsins are mycotoxins known to be responsible for fatal liver disease of lupin-fed sheep, and displayed phytotoxic and cytotoxic activities [206,207].

Ustiloxins (**286**–**291**) have a 13-membered ring including a phenol ether linkage. They were isolated from the water extract of the false smut balls caused by *Ustilaginoidea virens* on the panicles of rice plants [208,209,210,211,212]. There are three amino acids in the core ring for each ustiloxin. For ustiloxins C (**288**), D (**289**), F (**290**) and G (**291**), there is a glycine in the side chain. For ustiloxins A (**286**) and B (**287**), there are two amino acids in the side chains, whose tyrosine was modified with norvaline, a non-proteinogenic amino acid. They are biosynthesized by the ribosome, and are ribosomally synthesized and post-translationally modified peptides (RiPPs). The cluster possessed a gene, termed *ustA*, whose translated product, UstA, contained a 16-fold repeated peptide embedding a tetrapeptide, Tyr-Ala-Ile-Gly, that was converted into the cyclic moiety of ustiloxin B (**287**) [213]. Through gene inactivation, heterologous expression, and in vitro functional analyses the entire biosynthetic pathway of the ustiloxins was unveiled to involve at least nine enzymes. The oxidative cyclization of the core peptide is not clear yet though [214].

## 15. Conclusions and Future Perspectives

In this review, we describe the progress on the chemistry and biological activities of the cyclic peptides (excluding cyclic dipeptides and peptides containing ester bonds in the core ring) discovered from fungi during the past 50 years, especially the recent 20 years.

It seems that the fungi in genus *Aspergillus* can produce various types of cyclic peptides, especially cyclic tripeptides to pentapeptides, while the mushrooms, which we call macroscopic fungi, produce relatively big cyclic peptides such as amanin (an octapeptide), amanexitide (a nonapeptide), antamanide (decapeptide), omphalotins (dodecapeptide), and gymnopeptides (octadecapeptides). Most of cyclosporins, which belong to the cyclic undecapeptides, show immunosuppressive and antifungal activities. The isolated cyclic dodecapeptides have nematicidal activity, tetradecapeptides possess antibacterial activity, and octadecapeptides show antiproliferative activity on several human cancer cell lines. The cyclic peptides with a big ring seem to display more biological activities than the small-ring peptides.

Based on the biosynthetic machineries of fungal cyclic peptides, most of them belong are nonribosomal peptides (NRPs) which are synthesized by multimodular nonribosomal peptide synthetases (NRPSs). Just a few of them belong to the ribosomally synthesized and post-translationally modified peptides (RiPPs) such as α-amanitin (**218**) from *Amanita exitialis* [150], asperipin-2a (**279**) from *Aspergillus flavus* [203], phallacidin (**191**) from *Amanita bisporigera* [216], phomopsin A (**283**) from *Phomopsis leptostromiformis* [206], and ustiloxins (**286**-**291**) from *Ustilaginoidea virens* (teleomorph: *Villosiclva virens*) [208,209,212] and *Apsergillus flavus* [214], omphalotins (**260**–**268**) from *Omphalotus olearius* [194,195,196].

Some fungal cyclic peptides have potential to be developed into pharmaceuticals and agrochemicals. Some cyclic tetrapeptides such as 1-alaninechlamydocin (**24**) [30], apicidin (**25**) [35], AS1387392 (**36**) [37] and trapoxin A (**83**) [31] show inhibitory activity on histone deacetylase (HDAC) and have potential applications as therapeutics for spinal muscular atrophy [36], anti-cancer agents [30], antiprotozoal agents [35], and immunosuppressants [37]. Some cyclic peptides with phytotoxic activity show potential applications such as herbicides against weeds [217]. Biological analysis revealed a pro-apoptotic effect for most of the cyclic peptides that indicated that the cyclic peptides could be developed as the pro-apoptotic agents [218].

Some cyclic peptides have been developed as drugs, which are listed in Table 13. One noteworthy example is caspofungin which has strong antifungal activity. Caspofungin is a semisynthetic derivative of pneumocandin B_0_ (**170**), a lipophilic cyclic hexapeptide from the fungus *Glarea lozoyensis*. It was commercialized as the antifungal drug caspofungin acetate (CANCIDAS^®^) which has subsequently saved thousands of lives [14]. Micafungin was the second approved antifungal agent in the echinocandin series, and has been used worldwide in chemotherapy for life-threatening fungal infections. Micafungin is an inhibitor of 1,3-β-glucan synthase, an enzyme necessary for cell-wall synthesis of several fungal pathogens [121]. Other application examples included cyclosporin A (**238**), an immunosuppressant isolated from *Tolypocladium inflatum* [176]; omphalotin A (**264**), a potent nematicidal agent from *Omphalotus olearius* [197]; and the echinocandins, antifungal lipopeptides isolated from *Glarea lozoyensis*, *Coleophoma empetri*, and *Aspergillus nidulans* var. *echinulatus* [219]. Commercial cyclic peptide-derived drugs are listed in Table 14.

It is worth mentioning that many fungal cyclic peptides have been isolated from plant endophytic and marine-derived fungi, which indicates that plant endophytic and marine-drived fungi are mines of biologically active natural products [222,223,224,225,226]. Although the number of fungal cyclic peptides is gradually increasing, it remains a group of natural products with relatively little skeletal diversity. On many occasions, the relative or absolute configurations of the chiral centers have not been determined yet. This remains a challenge for future chemical and spectroscopic work. Many isolated cyclic peptides have not been screened for their biological activities as the quantities obtained were very limited. Even fewer compounds have been the subject of systematic investigations to establish structure-activity relationships. Further high throughput screening of biological activities as well as detailed studies on the action mechanisms of fungal cyclic peptides are needed.

## Figures and Tables

**Table 1:** Fungal cyclic tripeptides and their biological activities.

Name	Fungus and its Origin	Biological Activity	Ref.
Aspochracin (**1**)	*Aspergillus kumbius*	-	[24]
	*Aspergillus ochraceus*	Insecticidal activity	[17]
	*Aspergillus sclerotiorum* PT06-1	-	[19]
JBIR-15 (**2**)	*Aspergillus sclerotiorum*	Antifungal activity	[19,25]
Pre-sclerotiotide F (**3**)	*Aspergillus insulicola*	-	[26]
Psychrophilin A (**4**)	*Penicillium ribeum* from a soil under the tree *Ribes* sp.	-	[20]
Psychrophilin B (**5**)	*Penicillium ribeum* from a soil under the tree *Ribes* sp.	-	[21]
Psychrophilin C (**6**)	*Penicillium ribeum* from a soil under the tree *Ribes* sp.	-	[21]
Psychrophilin D (**7**)	*Penicillium algidum*	Cytotoxic activity	[18]
Psychrophilin E (**8**)	*Aspergillus versicolor* ZLN-60	Antiproliferative activity	[22,23]
Psychrophilin F (**9**)	*Aspergillus versicolor* ZLN-60	-	[23]
Psychrophilin G (**10**)	*Aspergillus versicolor* ZLN-60	Lipid-lowering effect	[23]
Psychrophilin H (**11**)	*Aspergillus versicolor* ZLN-60	-	[23]
Sclerotiotide A (**12**)	*Aspergillus sclerotiorum* PT06-1	Antifungal activity	[19]
Sclerotiotide B (**13**)	*Aspergillus sclerotiorum* PT06-1	Antifungal activity	[19]
Sclerotiotide C (**14**)	*Aspergillus sclerotiorum* PT06-1	-	[19]
Sclerotiotide D (**15**)	*Aspergillus sclerotiorum* PT06-1	-	[19]
Sclerotiotide E (**16**)	*Aspergillus sclerotiorum* PT06-1	-	[19]
Sclerotiotide F (**17**)	*Aspergillus sclerotiorum* PT06-1	Antifungal activity	[19]
	*Aspergillus insulicola*	-	[26]
Sclerotiotide G (**18**)	*Aspergillus sclerotiorum* PT06-1	-	[19]
Sclerotiotide H (**19**)	*Aspergillus sclerotiorum* PT06-1	-	[19]
Sclerotiotide I (**20**)	*Aspergillus sclerotiorum* PT06-1	Antifungal activity	[19]
Sclerotiotide J (**21**)	*Aspergillus sclerotiorum* PT06-1	-	[19]
Sclerotiotide K (**22**)	*Aspergillus sclerotiorum* PT06-1	-	[19]
Xyloallenolide A (**23**)	*Xylaria* sp. No. 2508	-	[27]

**Table 2:** Fungal cyclic tetrapeptides and their biological activities.

Name	Fungus and Its Origin	Biological Activity	Ref.
1-Alaninechlamydocin (**24**)	*Tolypocladium* sp.	Inhibitory activity on histone deacetyl (HDAC) activity, antiproliferation, cytotoxicity, cell cycle arrest, and apoptosis induction	[30]
Apicidin (**25**)	*Fusarium pallidoroseum*	Antimalarial activity	[32]
	*Fusarium semitectum*	Phytotoxic activity	[53]
	-	Antiprotozoal activity by inhibiting parasite HDAC	[35]
Apicidin A (**26**)	*Fusarium pallidoroseum*	Antimalarial activity	[32]
	*Fusarium semitectum*	-	[53]
Apicidin B (**27**)	*Fusarium pallidoroseum*	Antiprotozoal activity	[33]
Apicidin C (**28**)	*Fusarium pallidoroseum*	Antiprotozoal activity	[33]
Apicidin D_1_ (**29**)	*Fusarium pallidoroseum*	Antiprotozoal activity	[34]
Apicidin D_2_ (**30**)	*Fusarium pallidoroseum*	Antiprotozoal activity	[34]
	*Fusarium semitectum*	Phytotoxic activity	[53]
Apicidin D_3_ (**31**)	*Fusarium pallidoroseum*	Antiprotozoal activity	[34]
Apicidin E (**32**)	*Fusarium semitectum* mutant	-	[54]
Apicidin F (**33**)	*Fusarium fujikuroi*	Antimalarial activity	[55]
Apicidin G (**34**)	*Fusarium semitectum*	-	[56]
Apicidin H (**35**)	*Fusarium semitectum*	-	[56]
AS1387392 (**36**)	*Acremonium* sp. No. 27082	Immunosuppressive activity	[37]
Aspercolorin (**37**)	*Aspergillus versicolor*	-	[57]
Asperterrestide A (**38**)	*Aspergillus terreus* SCSGAF0162	Cytotoxic and antiviral activity	[38]
Auxarthride A (**39**)	Coprophilous fungus *Auxarthron pseudauxarthron*	-	[58]
Auxarthride B (**40**)	Coprophilous fungus *Auxarthron pseudauxarthron*	-	[58]
Chlamydocin (**41**)	*Diheterospora chlantydosporia*	Cytostatic activity	[39]
	*Penicillium* sp. BCC18034	Antimalarial and cytotoxic activity	[40]
Cyclo[(2*S*,9*R*)-9-(acetyloxy)-2-amino-8-oxodecanoyl-2-methylalanyl-l-phenylalanyl-d-prolyl] (**42**)	*Peniophora* sp.	Plant growth-retardant activity	[41]
Cyclo[2-methylalanyl-l-phenylalanyl-d-prolyl-(2*S*)-2-amino-8-oxodecanoyl] (**43**)	*Peniophora* sp.	Plant growth-retardant activity	[41]
Cyclo[2-methylalanyl-l-phenylalanyl-d-prolyl-(2*S*,9*R*)-2-amino-9-hydroxy-8-oxodecanoyl] (**44**)	*Peniophora* sp.	Plant growth-retardant activity	[41]
	*Verticillium coccosporum*	Phytotoxic activity	[59]
Cyclo(Gly-l-Phe-l-Pro-l-Tyr) (**45**)	Co-culture broth of two mangrove fungi *Phomopsis* sp. K38 and *Alternaria* sp. E33	Antifungal activity	[42]
Cyclo(l-Leu-*trans*-4-hydroxy-l-Pro-d-Leu-*trans*-4-hydroxy-l-Pro) (**46**)	Co-culture broth of two mangrove fungi *Phomopsis* sp. K38 and *Alternaria* sp. E33	Antifungal activity	[43]
Cyclo(d-Pro-l-Tyr-l-Pro-l-Tyr) (**47**)	Co-culture broth of two mangrove fungi *Phomopsis* sp. K38 and *Alternaria* sp. E33	Antifungal activity	[42]
Cyclo(*N*-methyl-l-Phe-l-Val-*N*-methyl-Phe-l-Val) (**48**)	*Onychocola sclerotica*	Cardiac calcium channel blocker	[44]
Cyclo(*N*-methyl-l-Phe-l-Val-*N*-methyl-l-Phe-l-Ile) (**49**)	*Onychocola sclerotica*	Cardiac calcium channel blocker	[44]
Cyclo(*N*-methyl-l-Phe-l-Ile-*N*-methyl-l-Phe-l-Ile) (**50**)	*Onychocola sclerotica*	Cardiac calcium channel blocker	[44]
Cyl-1 (**51**)	*Cylindrocladium scoparium*	Phytotoxic activity	[45,46]
Cyl-2 (**52**)	*Cylindrocladium scoparium*	Phytotoxic activity	[46,60]
Diheteropeptin (**53**)	*Diheterospora* sp.	TGF-β-like activity	[61]
	*Diheterospora chlamydosporia*	TGF-β-like activity	[62]
Dihydrotentoxin (**54**)	*Alternaria porri*	-	[63]
	*Phoma* sp.	-	[51]
Endolide A (**55**)	*Stachylideium* sp. from the sponge *Callyspongia* sp.	Affinity to the vaspopressin receptor 1A with a Ki of 7.04 μM	[64]
Endolide B (**56**)	*Stachylideium* sp. from the sponge *Callyspongia* sp.	Be selevtive toward the serotonin receptor 5HT2b with a Ki of 0.77 μM	[64]
Endolide C (**57**)	Marine-sponge-derived *Stachylidium* sp. 293 K04	-	[65]
Endolide D (**58**)	Marine-sponge-derived *Stachylidium* sp. 293 K04	-	[65]
5,5’-Epoxy-MKN-349A (**59**)	Endophytic fungus *Penicillium* sp. GD6 from the mangrove *Bruguiera gymnorrhiza*	-	[66]
FR235222 (**60**)	*Acremonium* sp. No. 27082	Immunosuppressant that inhibits mammalian histone deacetylase	[67,68,69]
Fungisporin (**61**)	*Penicillium chrysogenum*	-	[70]
HC-toxin (**62**)	*Helminthosporium carbonum* = *Cochliobolus carbonum*	Phytotoxic activity	[71,72]
Hirsutide (**63**)	Spider-derived fungus *Hirsutella* sp.	-	[73]
Isotentoxin (**64**)	*Alternaria porri*	-	[63]
JM47 (**65**)	Marine-derived *Fusarium* sp. from the alga *Codium fragile*	-	[29]
Microsporin A (**66**)	*Microsporum* cf. *gypseum* CNL-692	Inhibitory activity on histone deacetylase; cytotoxic activity	[47]
Microsporin B (**67**)	*Microsporum* cf. *gypseum* CNL-692	Inhibitory activity on histone deacetylase; cytotoxic activity	[47]
Nidulanin A (**68**)	*Aspergillus nidulans*	-	[74]
Penicopeptide A (**69**)	Endophytic *Penicillium commune* from *Vitis vinifera*	Inhibitrory activity on 11β-hydroxysteroid dehydrogense	[48]
PF1070A (**70**)	*Calonectria ilicicola*	Phytotoxic activity	[75]
	*Humicola* sp.	Antitumour activity	[76]
PF1070B (**71**)	*Humicola* sp.	Antitumour activity	[76]
Phoenistatin (**72**)	*Acremonium fusigerum*	Enhancing activity on gene expression	[77]
Pseudoxylallemycin A (**73**)	Termite-associated fungus *Pseudoxylaria* sp. X802	Antibacterial and antiproliferative activities	[49]
Pseudoxylallemycin B (**74**)	Termite-associated fungus *Pseudoxylaria* sp. X802	Antibacterial and antiproliferative activities	[49]
Pseudoxylallemycin C (**75**)	Termite-associated fungus *Pseudoxylaria* sp. X802	Antibacterial and antiproliferative activities	[49]
Pseudoxylallemycin D (**76**)	Termite-associated fungus *Pseudoxylaria* sp. X802	Antibacterial and antiproliferative activities	[49]
Pseudoxylallemycin E (**77**)	Termite-associated fungus *Pseudoxylaria* sp. X802	-	[49]
Pseudoxylallemycin F (**78**)	Termite-associated fungus *Pseudoxylaria* sp. X802	-	[49]
Sartoryglabramide A (**79**)	Marine-derived *Neosartorya glabra* KUFA 0702 from the sponge *Mycale* sp.	-	[78]
Sartoryglabramide B (**80**)	Marine-derived *Neosartorya glabra* KUFA 0702 from the sponge *Mycale* sp.	-	[78]
Tentoxin (**81**)	*Alternaria linicola*	Phytotoxic activity	[79]
	*Alternaria porri*	-	[63]
	*Phoma* sp.	-	[51]
Tentoxin B (**82**)	Marine-derived fungus *Phoma* sp. from the giant jellyfish *Nemopilema nomurai*	Weak suppressive effect on the production of nitric oxide in murine macrophage cells without notable cytotoxicity	[51]
Trapoxin A (**83**)	*Helicoma ambiens* RF-1023	Detransformation activity against *v-sis* oncogene-transformed NIH3T3 cells	[52]
	*-*	HDAC inhibitory activity	[31]
Trapoxin B (**84**)	*Helicoma ambiens* RF-1023	Detransformation activity against *v-sis* oncogene-transformed NIH3T3 cells	[52]
WF-3161 (**85**)	*Petriella guttlata*	Antitumor activity	[80]

**Table 3:** Fungal cyclic pentapeptides and their biological activities.

Name	Fungus and Its Origin	Biological Activity	Ref.
Argadin (**86**)	*Clonostachys* sp. FO-7314	Chitinase inhibitor	[81]
Argifin (**87**)	*Gliocladium* sp. FTD-0668	Chitinase inhibitor	[82,97]
Aspergillipeptide D (**88**)	Marine gorgonian-derived fungus *Aspergillus* sp. SCSIO 41501	Antiviral activity	[84]
Asperpeptide A (**89**)	Marine gorgonian-derived *Aspergillus* sp. XS-20090B15	Antibacterial activity	[98]
Avellanin A (**90**)	*Hamigera* sp.	Vasoconstriction and pressor effect	[99]
Avellanin B (**91**)	*Hamigera* sp.	Vasoconstriction and pressor effect	[99]
Avellanin C (**92**)	*Hamigera paravellanea*	Vasoconstriction and pressor effect	[99]
Chrysosporide (**93**)	*Sepedonium chrysospermum*	Weak cytotoxic to murine leukemia cell line	[100]
Clavatustide C (**94**)	*Aspergillus clavatus* C2WU	-	[101]
Cotteslosin A (**95**)	*Aspergillus versicolor*	Weak cytotoxic to human cancer cell lines	[102]
Cotteslosin B (**96**)	*Aspergillus versicolor*	Weak cytotoxic to human cancer cell lines	[102]
Cyclo(l-Ile-l-Leu-l-Leu-l-Leu-l-Leu) (**97**)	Endophytic fungus *Cryptosporiopsis* sp. of *Zanthoxylum leprieurii*	Motility inhibitory and lytic activities against zoospores; Antifungal activity; weak cytotoxic activity on brine shrimp larvae	[103]
Cyclo(l-Leu-l-Leu-d-Leu-l-Leu-l-Ile) (**98**)	Endophytic fungus *Fusarium decemcellulare* LG53 from Chinese medicinal plant *Mahonia fortunei*	Antagonistic effect on fungus	[104]
Cyclo(l-Leu-l-Leu-d-Leu-l-Leu-l-Leu) (**99**)	Endophytic fungus *Fusarium decemcellulare* LG53 from Chinese medicinal plant *Mahonia fortunei*	Antagonistic effect on fungus	[104]
Cyclo(l-Leu-l-Leu-d-Leu-l-Leu-l-Val) (**100**)	Endophytic fungus *Fusarium decemcellulare* LG53 from Chinese medicinal plant *Mahonia fortunei*	Antagonistic effect on fungus	[104]
Cyclo(l-Phe-l-Leu-l-Leu-l-Leu-l-Ile) (**101**)	Unidentified endophytic fungus No. 2524 from the mangrove *Avicennia marina*	-	[105]
Cyclo(l-Phe-l-Leu-l-Leu-l-Leu-l-Leu) (**102**)	Endophytic fungus *Cryptosporiopsis* sp. of *Zanthoxylum leprieurii*	Motility inhibitory and lytic activities against zoospores; Antifungal activity; Weak cytotoxic activity on brine shrimp larvae	[103]
Cycloaspeptide A (**103**)	*Aspergillus* sp. NE-45	-	[85]
	*Penicillium algidum*	Antiplasmodial activity	[18]
	*Penicillium jamesonlandense*	-	[106]
	*Penicillium janczewskii*	-	[107]
	*Penicillium lanosum*	-	[106]
	*Penicillium ribium*	-	[106]
	*Penicillium ribeum* from a soil under the tree *Ribes* sp.	-	[20]
	*Penicillium soppii*	-	[106]
Cycloaspeptide B (**104**)	*Aspergillus* sp. NE-45	-	[85]
Cycloaspeptide C (**105**)	*Aspergillus* sp. NE-45	-	[85]
Cycloaspeptide D (**106**)	*Penicillium algidum*	Antiplasmodial activity	[18]
	*Penicillium ribeum* from a soil under the tree *Ribes* sp.	-	[20]
Cycloaspeptide E (**107**)	*Penicillia* sp.	Insecticidal activity	[86]
	*Tricothecium* sp.	-	[86]
Cycloaspeptide F (**108**)	*Isaria farinosa*	Cytotoxic activity	[87]
Cycloaspeptide G (**109**)	*Isaria farinosa*	Cytotoxic activity	[87]
Cyclochlorotine (**110**)	*Penicillium islandicum*	Liver fibrosis, liver injuries	[108,109,110]
Hydroxycyclochlorotine (**111**)	*Penicillium islandicum*	-	[108]
Islanditoxin (**112**)	*Penicillium islandicum*	-	[111]
Lajollamide A (**113**)	Marine-derived fungus *Asteromyces cruciatusis*	Weak antibacterial on Gram-positive bacteria	[112]
Malformin A1 (**114**)	*Aspergillus niger*	Malformation activity in the corn root; Cytotoxic activity	[92,93]
	*Aspergillus tubingensis*	Cytotoxic activity; Anti-TMV activity	[89,90]
Malformin A2 (**115**)	*Aspergillus niger*	-	[92]
Malformin B1a (**116**)	*Aspergillus niger*	Curvature activity in the corn root test	[88]
Malformin B1b (**117**)	*Aspergillus niger*	-	[88]
Malformin B2 (**118**)	*Aspergillus niger*	-	[88]
Malformin C (**119**)	*Aspergillus niger*	Anticancer activity	[94,95]
Malformin E (**120**)	Endophytic fungus *Aspergillus tamari* from *Ficus carica*	Cytotoxic and antibiotic activity	[91]
MBJ-0174 (**121**)	*Mortierella alpine* f28740 from a soil smaple collected in Ise, Japan	Stimulators of cellular fibrinolytic activity	[113]
PF1171B (**122**)	*Hamigera* sp.	-	[99]
PF1171D (**123**)	*Hamigera* sp.	-	[99]
PF1171E (**124**)	*Hamigera* sp.	-	[99]
Pseudacyclin A (**125**)	Animal fungal pathogen *Pseudallescheria boydii*	Immunosuppressive activity	[96]
Pseudacyclin B (**126**)	Animal fungal pathogen *Pseudallescheria boydii*	-	[96]
Pseudacyclin C (**127**)	Animal fungal pathogen *Pseudallescheria boydii*	-	[96]
Pseudacyclin D (**128**)	Animal fungal pathogen *Pseudallescheria boydii*	-	[96]
Pseudacyclin E (**129**)	Anmal fungal pathogen *Pseudallescheria boydii*	-	[96]
Versicoloritide A (**130**)	*Aspergillus versicolor* LCJ-5-4	-	[114]
Versicoloritide B (**131**)	*Aspergillus versicolor* LCJ-5-4	-	[114]
Versicoloritide C (**132**)	*Aspergillus versicolor* LCJ-5-4	-	[114]
Versicotide A (**133**)	*Aspergillus versicolor* ZLN-60 from the sediment of the Yellow Sea	-	[115]
Versicotide B (**134**)	*Aspergillus versicolor* ZLN-60 from the sediment of the Yellow Sea	-	[115]
Xylarotide A (**135**)	*Xylaria* sp. 101	-	[116]
Xylarotide B (**136**)	*Xylaria* sp. 101	-	[116]

**Table 4:** Fungal cyclic hexapeptides and their biological activities.

Name	Fungus and Its Origin	Biological Activity	Ref.
Aculeacin A (**137**)	*Aspergillus aculeatus* M-4214	Antifungal activity by inhibiting synthesis of β-1,3-glucan	[117,118]
Anthranicine (**138**)	*Acremonium* sp. A29-2004	-	[130]
ASP2397 (**139**)	*Acremonium persicinum* MF-347833 from Malaysian leaf litter	Antifungal activity	[131]
AS2524371 (**140**)	*Acremonium persicinum* MF-347833 from Malaysian leaf litter	-	[131]
AS2488059 (**141**)	*Acremonium persicinum* MF-347833 from Malaysian leaf litter	-	[131]
Cryptocandin (**142**)	*Cryptosporiopsis* cf. *quercina*	Antifungal activity	[132]
Deoxymulundocandin (**143**)	*Aspergillus sydowii*	Antifungal activity	[133]
	*Aspergillus mulundensis*	Antifungal activity	[123]
Echinocandin B (**144**)	*Aspergillus nidulans* var. *roseus* ATCC 58397	Antifungal activity	[134]
	*Aspergillus mulundensis*	Antifungal activity	[123]
Echinocandin C (**145**)	*Aspergillus* sp.	Antifungal activity	[135]
	*Aspergillus mulundensis*	Antifungal activity	[123]
Echinocandin D (**146**)	*Aspergillus* sp.	Antifungal activity	[135]
Ferrichrome (**147**)	*Ustilago maydis*	-	[136]
Ferrichrome A (**148**)	*Ustilago maydis*	-	[136]
Ferricrocin (**149**)	*Colletotrichum gloeosporioides*	Phytotoxic activity	[137]
Ferrirhodin (**150**)	*Botrytis cinerea*	-	[138]
FR190293 (**151**)	*Tolypocladium parasiticum* No. 16616	Antifungal activity	[139]
FR209602 (**152**)	*Coleophoma crateriformis* No. 738	Antifungal activity	[140]
FR209603 (**153**)	*Coleophoma crateriformis* No. 738	Antifungal activity	[140]
FR209604 (**154**)	*Coleophoma crateriformis* No. 738	Antifungal activity	[140]
FR220897 (**155**)	*Coleophoma empetri* No. 14573	Antifungal activity	[141]
FR220899 (**156**)	*Coleophoma empetri* No. 14573	Antifungal activity	[141]
FR227673 (**157**)	*Chalara* sp. No. 22210	Antifungal activity	[139]
FR901379 (**158**)	*Coleophoma empetri* F-11899	Antifungal activity	[142]
Mulundocandin (**159**)	*Aspergillus sydowii*	Antifungal activity	[122]
	*Aspergillus mulundensis*	Antifungal activity	[123]
Penitropeptide (**160**)	Endophytic fungus *Penicillium tropicum* isolated from leaves of *Sapium ellipticum*	-	[143]
PF1171A (**161**)	Unidentified ascomycete OK-128	Paralytic activity against silkworms	[124]
PF1171C = Similanamide (**162**)	Marine sponge-associated fungus *Aspergillus similanensis* KUFA 0013	Weak cytotoxic activity against the cancer cell lines	[125,126]
	*Hamigera* sp.	-	[99]
	Unidentified ascomycete OK-128	Paralytic activity against silkworms	[124]
PF1171F (**163**)	Unidentified ascomycete OK-128	Paralytic activity against silkworms	[144]
PF1171G (**164**)	Unidentified ascomycete OK-128	Paralytic activity against silkworms	[144]
Pneumocandin A_0_ = L-671,329 (**165**)	*Zalerion arboricola*	Antipneumocystis, anticandida and *Candida albicans* 1,3-β-d-glucan synthesis inhibition activity	[127,128]
	*Zalerion arboricola*	Antifungal activity	[145]
Pneumocandin A_1_ (**166**)	*Zalerion arboricola*	Anticandida activity and *Candida albicans* 1,3-β-d-glucan synthesis inhibition activity	[127,128]
Pneumocandin A_2_ (**167**)	*Zalerion arboricola*	Anticandida activity and *Candida albicans* 1,3-β-d-glucan synthesis inhibition activity	[127,128]
Pneumocandin A_3_ (**168**)	*Zalerion arboricola*	Anticandida activity and *Candida albicans* 1,3-β-d-glucan synthesis inhibition activity	[127,128]
Pneumocandin A_4_ (**169**)	*Zalerion arboricola*	Anticandida activity and *Candida albicans* 1,3-β-d-glucan synthesis inhibition activity	[127,128]
Pneumocandin B_0_ (**170**)	*Zalerion arboricola*	Antipneumocystis, anticandida and *Candida albicans* 1,3-β-d-glucan synthesis inhibition activity	[127,128]
Pneumocandin B_2_ (**171**)	*Zalerion arboricola*	Antipneumocystis, anticandida and *Candida albicans* 1,3- β-d-glucan synthesis inhibition activity	[127,128]
Pneumocandin C_0_ (**172**)	*Zalerion arboricola*	Antipneumocystis, anticandida and *Candida albicans* 1,3- β-d-glucan synthesis inhibition activity	[127,128]
Sclerotide A (**173**)	*Aspergillus sclerotiorum* PT06-1	Antifungal activity	[129]
Sclerotide B (**174**)	*Aspergillus sclerotiorum* PT06-1	Antifungal, antibacterial and cytotoxic activity	[129]
Simplicilliumtide M (**175**)	Deep-sea derived *Simplicillium obclavatum* EIODSF 020	-	[146]
Versicotide C (**176**)	*Aspergillus versicolor* ZLN-60	-	[23]

**Table 5:** Fungal cyclic heptapeptides and their biological activities.

Name	Fungus and Its Origin	Biological Activity	Ref.
Aladesoxiviroidin (**177**)	*Amanita phalloides*	Toxic to some animal species	[150]
Alaviroidin (**178**)	*Amanita phalloides*	Toxic to some animal species	[150]
Cordyheptapeptide A (**179**)	*Cordyceps* sp. BCC1788	Antimalarial activity against *Plasmodium falciparum* K1	[147]
	*Cordyceps* sp. BCC 16176	Cytotoxic activity	[148]
Cordyheptapeptide B (**180**)	*Cordyceps* sp. BCC 16176	Cytotoxic activity	[148]
Cordyheptapeptide C (**181**)	*Acremonium persicinum* SCSIO 115	Cytotoxic activity	[149]
Cordyheptapeptide D (**182**)	*Acremonium persicinum* SCSIO 115	-	[149]
Cordyheptapeptide E (**183**)	*Acremonium persicinum* SCSIO 115	Cytotoxic activity	[149]
Deoxoviroidin (**184**)	Mushroom *Amanita virosa*	Hepatotoxicity	[159]
Deoxoviroisin (**185**)	Mushroom *Amanita virosa*	Hepatotoxicity	[159]
Epichloenin B (**186**)	*Epichloe festucae*	-	[160]
Mortiamide A (**187**)	Marine-derived *Mortierella* sp.	-	[161]
Mortiamide B (**188**)	Marine-derived *Mortierella* sp.	-	[161]
Mortiamide C (**189**)	Marine-derived *Mortierella* sp.	-	[161]
Mortiamide D (**190**)	Marine-derived *Mortierella* sp.	-	[161]
Phallacidin (**191**)	*Amanita phalloides*	Toxic to some animal species	[150]
Phallacin (**192**)	*Amanita phalloides*	Toxic to some animal species	[150]
Phallisacin (**193**)	*Amanita phalloides*	Toxic to some animal species	[150]
Phallisin (**194**)	*Amanita phalloides*	Toxic to some animal species	[150]
Phalloidin (**195**)	*Amanita phalloides*	Toxic to some animal species	[150]
Phalloin (**196**)	*Amanita phalloides*	Toxic to some animal species	[150]
Prophallin (**197**)	*Amanita phalloides*	Toxic to some animal species	[150]
Scytalidamide A (**198**)	*Acremonium* sp.	-	[152]
	*Scytalidiu* sp.	Cytotoxic activity on HCT-116	[153]
Scytalidamide B (**199**)	*Acremonium* sp.	-	[152]
	*Scytalidiu* sp.	Cytotoxic activity on HCT-116	[153]
Serinocyclin A (**200**)	*Metarhizium anisopliae*	Insecticidal activity on mosquito larvae	[162]
Serinocyclin B (**201**)	*Metarhizium anisopliae*	-	[162]
Talarolide A (**202**)	Marine tunicte-associated fungus *Talaromyces* sp. CMB-TU011	-	[163]
Talaromin A (**203**)	*Talaromyces wortmannii*	-	[164]
Talaromin B (**204**)	*Talaromyces wortmannii*	-	[164]
Ternatin (**205**)	*Cladobotryum* sp.	Cytotoxic activity; antifungal activity	[165]
Unguisin A (**206**)	Marine-derived *Emericella unguis*	Moderate antibacterial activity	[154,155]
Unguisin B (**207**)	Marine-derived *Emericella unguis*	Moderate antibacterial activity	[154,155]
Unguisin C (**208**)	Marine-derived *Emericella unguis*	-	[155]
Unguisin D (**209**)	Marine-derived *Emericella unguis*	-	[155]
Unguisin E (**210**)	*Aspergillus* sp. AF119	-	[156]
	Endophytic fungus *Mucor irregularis* from the medicinal plant *Moringa stenopetala*	-	[157]
Unguisin F (**211**)	Endophytic fungus *Mucor irregularis* from the medicinal plant *Moringa stenopetala*	-	[157]
Viroidin (**212**)	Mushroom *Amanita virosa*	-	[159]
	*Amanita phalloides*	Toxic to some animal species	[150]
Viroisin (**213**)	Mushroom *Amanita virosa*	-	[159]

**Table 6:** Fungal cyclic octapeptides and their biological activities.

Name	Fungus and Its Origin	Biological Activity	Ref.
Amanin (**214**)	*Amanita phalloides*	Toxic to some animal species	[150]
Amanin amide (**215**)	*Amanita phalloides*	Toxic to some animal species	[150]
Amanullin (**216**)	*Amanita phalloides*	Toxic to some animal species	[150]
Amanullinic acid (**217**)	*Amanita phalloides*	Toxic to some animal species	[150]
α-Amanitin (**218**)	*Amanita exitialis*	-	[168]
	*Amanita subjunquillea*	Cytotoxic activity	[169]
	*Amanita phalloides*	Toxic to some animal species	[150]
β-Amanitin (**219**)	*Amanita exitialis*	-	[168]
	*Amanita subjunquillea*	Cytotoxic activity	[169]
	*Amanita phalloides*	Toxic to some animal species	[150]
γ-Amanitin (**220**)	*Amanita phalloides*	Toxic to some animal species	[150]
ε-Amanitin (**221**)	*Amanita phalloides*	Toxic to some animal species	[150]
Epichlicin (**222**)	Endophytc *Epichloe typhina* from *Phleum pratense*	Inhibitory activity on the spore germination of *Cladosporium phlei*	[166]
Epichloeamide (**223**)	*Epichloe festucae*	-	[160]
Epichloenin A (**224**)	*Epichloe festucae*	-	[160]
Proamanullin (**225**)	*Amanita phalloides*	Toxic to some animal species	[150]
Shearamide A (**226**)	*Eupenicillium shearii*	Insecticidal effect	[167]

**Table 7:** Fungal cyclic nonapeptides and their biological activities.

Name	Fungus and Its Origin	Biological Activity	Ref.
Amanexitide (**227**)	*Amanita exitialis*	-	[168]
Clonostachysin A (**228**)	Marine-derived fungus *Clonostachys rogersoniana* HJK9 from sponge	Anti-dinoflagellate activity	[170]
Clonostachysin B (**229**)	Marine-derived fungus *Clonostachys rogersoniana* HJK9 from sponge	Anti-dinoflagellate activity	[170]
Cylindrocyclin A (**230**)	*Cylindrocarpon* sp.	Cytotoxic activity	[171]

**Table 8:** Fungal cyclic decapeptides and their biological activities.

Name	Fungus and Its Origin	Biological Activity	Ref.
Antamanide (**231**)	*Amanita phalloides*	Inhibition of the mitochondrial permeability transition pore, antidote activity against phallotoxins and amatoxins, inhibition of tumor cell growth in vitro	[172,175]
Arborcandin A (**232**)	Unidentified fungus SANK 17397	Inhibitory activity on 1,3-β-glucan synthase	[173,174]
Arborcandin B (**233**)	Unidentified fungus SANK 17397	Inhibitory activity on 1,3-β-glucan synthase	[173,174]
Arborcandin C (**234**)	Unidentified fungus SANK 17397	Inhibitory activity on 1,3-β-glucan synthase	[173,174]
Arborcandin D (**235**)	Unidentified fungus SANK 17397	Inhibitory activity on 1,3-β-glucan synthase	[173,174]
Arborcandin E (**236**)	Unidentified fungus SANK 17397	Inhibitory activity on 1,3-β-glucan synthase	[173,174]
Arborcandin F (**237**)	Unidentified fungus SANK 17397	Inhibitory activity on 1,3-β-glucan synthase	[173,174]

**Table 9:** Fungal cyclic undecapeptides and their biological activities.

Name	Fungus and Its Origin	Biological Activity	Ref.
Cyclosporin A (**238**)	*Aspergillus fumigatus*	-	[177]
	*Aspergillus terreus* NRC FA 200	-	[178]
	*Beauveria nivea*	-	[179]
	*Fusarium oxysporum*	Immunosuppressive, antifungal, anti-inflammatory, anti-parasitic and anticancer activities	[180]
	*Tolypocladium inflatum*	-	[182]
	*Tolypocladium* sp.	-	[183]
	*Trichoderma polysporum*	Antifungal activity	[181]
Cyclosporin B (**239**)	*Tolypocladium inflatum*	Immunosuppressive and antifungal activities	[182,186]
	*Trichoderma polysporum*	Antifungal activity	[181]
Cyclosporin C (**240**)	*Acremonium luzulae*	Antifungal activity	[187]
	*Tolypocladium inflatum*	Immunosuppressive and antifungal activities	[182,186]
Cyclosporin D (**241**)	*Tolypocladium inflatum*	Immunosuppressive and antifungal activities	[182,186]
	*Trichoderma polysporum*	Antifungal activity	[181]
Cyclosporin E (**242**)	*Trichoderma polysporum*	-	[181]
	*Tolypocladium inflatum*	Immunosuppressive and antifungal activities	[186,188]
Cyclosporin F (**243**)	*Tolypocladium inflatum*	Immunosuppressive and antifungal activities	[186,188]
Cyclosporin G (**244**)	*Tolypocladium inflatum*	Immunosuppressive and antifungal activities	[182,186,188]
Cyclosportin H (**245**)	*Tolypocladium inflatum*	-	[188]
Cyclosportin I (**246**)	*Tolypocladium inflatum*	Immunosuppressive and antifungal activities	[186,188]
Cyclosportin J (**247**)	*Tolypocladium terricola*	Increased cell proliferation	[189]
Cyclosporin K (**248**)	*Tolypocladium inflatum*	Immunosuppressive and antifungal activities	[186]
Cyclosporin L (**249**)	*Tolypocladium inflatum*	Immunosuppressive and antifungal activities	[186]
Cyclosporin M (**250**)	*Tolypocladium inflatum*	Immunosuppressive and antifungal activities	[186]
Cyclosporin N (**251**)	*Tolypocladium inflatum*	Immunosuppressive and antifungal activities	[186]
Cyclosporin O (**252**)	*Tolypocladium inflatum*	Immunosuppressive and antifungal activities	[186]
Cyclosporin P (**253**)	*Tolypocladium inflatum*	Immunosuppressive and antifungal activities	[186]
Cyclosporin Q (**254**)	*Tolypocladium inflatum*	Immunosuppressive and antifungal activities	[186]
Cyclosporin S (**255**)	*Tolypocladium inflatum*	Immunosuppressive and antifungal activities	[186]
Cyclosporin T (**256**)	*Epichloe bromicola*	Antifungal activity	[190]
	*Tolypocladium inflatum*	Immunosuppressive and antifungal activities	[186]
Cyclosporin U (**257**)	*Tolypocladium inflatum*	Immunosuppressive and antifungal activities	[186]
Cyclosporin V (**258**)	*Tolypocladium inflatum*	Immunosuppressive and antifungal activities	[186]
Cyclosporin W (**259**)	*Tolypocladium inflatum*	Immunosuppressive and antifungal activities	[186]
Cyclosporin X (**260**)	*Tolypocladium inflatum*	Immunosuppressive and antifungal activities	[186]
Cyclosporin Y (**261**)	*Tolypocladium inflatum*	Immunosuppressive and antifungal activities	[186]
Cyclosporin Z (**262**)	*Tolypocladium inflatum*	Immunosuppressive and antifungal activities	[186]
FR901459 (**263**)	*Stachybotrys chartarum* No. 19392 from a soil collected in Tokyo, Japan	Immunosuppressive activity	[184]

**Table 10:** Fungal cyclic dodecapeptides and their biological activities.

Name	Fungus and Its Origin	Biological Activity	Ref.
Omphalotin A = Omphalotin (**264**)	Mushroom *Omphalotus olearius*	Nematicidal activity	[191,197,198,199]
Omphalotin B (**265**)	Mushroom *Omphalotus olearius*	Nematicidal activity	[191]
Omphalotin C (**266**)	Mushroom *Omphalotus olearius*	Nematicidal activity	[191]
Omphalotin D (**267**)	Mushroom *Omphalotus olearius*	Nematicidal activity	[191]
Omphalotin E (**268**)	Mushroom *Omphalotus olearius*	Nematicidal activity on *Meloidogyne incognita*	[192]
Omphalotin F (**269**)	Mushroom *Omphalotus olearius*	Nematicidal activity on *Meloidogyne incognita*	[192]
Omphalotin G (**270**)	Mushroom *Omphalotus olearius*	Nematicidal activity on *Meloidogyne incognita*	[192]
Omphalotin H (**271**)	Mushroom *Omphalotus olearius*	Nematicidal activity on *Meloidogyne incognita*	[192]
Omphalotin I (**272**)	Mushroom *Omphalotus olearius*	Nematicidal activity on *Meloidogyne incognita*	[192]

**Table 11:** Fungal cyclic tetradecapeptides and their biological activities.

Name	Fungus and Its Origin	Biological Activity	Ref.
Verrucamide A (**273**)	Plant pathogenic fungus *Myrothecium verrucaria* on crops and weeds	Antibacterial activity on *Staphylococcus aureus*	[200]
Verrucamide B (**274**)	Plant pathogenic fungus *Myrothecium verrucaria* on crops and weeds	Antibacterial activity on *Staphylococcus aureus*	[200]
Verrucamide C (**275**)	Plant pathogenic fungus *Myrothecium verrucaria* on crops and weeds	Antibacterial activity on *Staphylococcus aureus*	[200]
Verrucamide D (**276**)	Plant pathogenic fungus *Myrothecium verrucaria* on crops and weeds	Antibacterial activity on *Staphylococcus aureus*	[200]

**Table 12:** Fungal cyclic octadecapeptides and their biological activities.

Name	Fungus and its Origin	Biological Activity	Ref.
Gymnopeptide A (**277**)	Mushroom *Gymnopus fusipes*	Antiproliferative activity on several human cancer cell lines	[201]
Gymnopeptide B (**278**)	Mushroom *Gymnopus fusipes*	Antiproliferative activity on several human cancer cell lines	[201]

**Table 13:** Fungal cyclic peptides containing ether bonds in the core ring and their biological activities.

Name	Fungus and Its Origin	Biological Activity	Ref.
Asperipin-2a (**279**)	*Aspergillus flavus*	-	[203]
*S*-Deoxyustiloxin H (**280**)	*Aspergillus flavus*	-	[214]
*N*-Desmethylustiloxin F (**281**)	*Aspergillus flavus*	-	[214]
HV-toxin M (**282**)	Plant pathogenic fungus *Cochliobus victoriae = Helminthosporium victoria* from oat	Phytotoxic activity	[205]
Phomopsin A (**283**)	Plant pathogenic fungus *Phomopsis leptostromiformis* from lupinis (*Lupinus* spp.)	Phytotoxic activity, toxic to animals	[206]
Phomopsin B (**284**)	Plant pathogenic fungus *Phomopsis leptostromiformis* from lupinis (*Lupinus* spp.)	Toxic to animals	[207]
Phomopsin F (**285**)	Plant pathogenic fungus *Diaporthe toxica*	Cytotoxic activity against Hepg2 cells	[208]
Ustiloxin A (**286**)	Plant pathogenic fungus *Ustilaginoidea virens* from rice false smut balls	Cytotoxic, phytotoxic activities	[209,211]
Ustiloxin B (**287**)	Plant pathogenic fungus *Ustilaginoidea virens* from rice false smut balls	Cytotoxic, phytotoxic activities	[209,211]
	*Aspergillus flavus*	-	[213]
Ustiloxin C (**288**)	Plant pathogenic fungus *Ustilaginoidea virens* from rice false smut balls	Cytotoxic, phytotoxic activities	[208]
Ustiloxin D (**289**)	Plant pathogenic fungus *Ustilaginoidea virens* from rice false smut balls	Cytotoxic, phytotoxic activities	[208]
Ustiloxin F (**290**)	Plant pathogenic fungus *Ustilaginoidea virens* from rice false smut balls	Cytotoxic, phytotoxic activities	[209]
Ustiloxin G (**291**)	Plant pathogenic fungus *Ustilaginoidea virens* from rice false smut balls	Cytotoxic, phytotoxic activities	[212]
Ustiloxin H (**292**)	*Aspergillus flavus*	-	[214]
Victorin C (**293**)	Plant pathogenic fungus *Cochliobus victoriae = Helminthosporium victoria*	Phytotoxic activity	[215]

**Table 14:** Some examples of the commercial drugs developed from fungal cyclic peptides.

Product Name	Original Cyclic Peptide	Development Way	Application	Ref.
Anidulafungin	Echinocandin B (**144**)	Semisynthesis	Antifungal agent	[220]
CANCIDAS^®^	Pneumocandin B_0_ (**170**)	Semisynthesis	Antifungal agent	[14]
Caspofungin	Echinocandins	Direct application	Antifungal agent for many *Candida* infections	[120]
Cyclosporin A	Cyclosporin A (**238**)	Direct application	Immunosuppressive and antifungal drug	[176]
Micafungin	Echinocandins	Semisynthesis	Antifugal agent	[221]
